# r^2^SCAN-Based Composite Methods for Solid-State Calculations

**DOI:** 10.1021/acs.jctc.6c00852

**Published:** 2026-08-29

**Authors:** Mattia Raimondo, Lorenzo Donà, Bartolomeo Civalleri

**Affiliations:** Dipartimento di Chimica, 9314 Università di Torino and NIS interdepartmental center, Via P. Giuria 7, 10125 Torino, Italy

## Abstract

In this work, we have extended the existing “sol-3c” composite methods to meta-GGA XC functionals using the r^2^SCAN one. The corresponding r^2^SCAN-based composite methods in their pure and hybrid HF/DFT variants have been developed in combination with double-ζ (sol-def2-mSVP) and triple-ζ (pob-TZVP-rev2) quality basis sets specifically adapted to solids. The r^2^SCAN “sol-3c/pob-3c” methods are validated and tested on benchmark sets containing both intermolecular adducts (S66 × 7), molecular crystals (X23, DMC8) and simple inorganic solids (SS20). They are then applied to selected applications of relevance for solid-state calculations such as the study of the energetic and structural properties of layered materials, the mechanical, electronic and vibrational properties of α-quartz, relative stability of all-silica zeolite polymorphs, α-quartz vs stishovite phase transition and the adsorption of small molecules on the surface of inorganic solids and in the pore of metal–organic frameworks. The new methods show broad applicability with accurate results and an adjustable and affordable computational cost.

## Graphical abstract

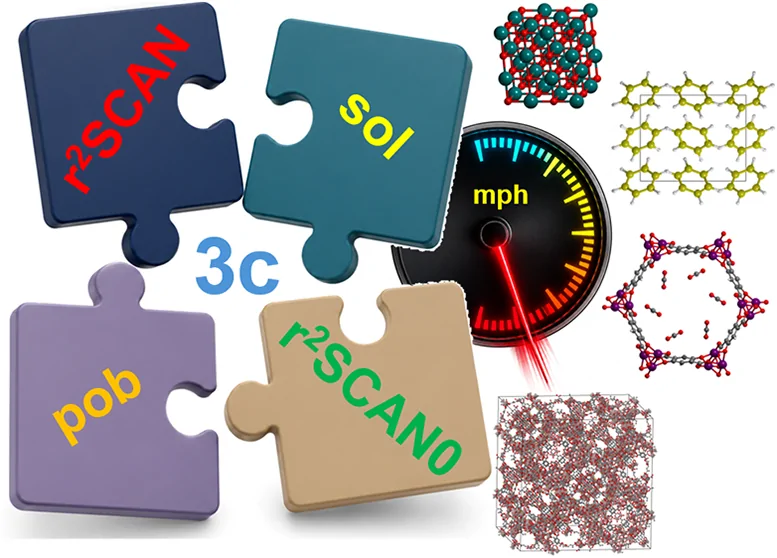

## 1 Introduction

In the last decades, methods derived from Density Functional Theory (DFT) have been used as workhorse computational tools for many applications.^1–3^ Recently, there has been a growing interest in moving from the modeling of small and medium-sized systems to investigating increasingly large and complex systems. However, for systems characterized by weak bonds, the current semilocal or hybrid DFT methods do not show sufficient accuracy and predictive power due to their pitfall in describing dispersive interactions.^4–6^ To cope with this, it would be necessary to use either the fifth-rung exchange-correlation (XC) functionals or wave function-based methods, which are very accurate but still extremely expensive for large-scale calculations. To overcome this limitation, Stefan Grimme and co-workers proposed the so-called “3c” composite methods which combine various DF approximations with semiclassical corrections to include dispersive interactions and correct for the basis set superposition error of the adopted small-to-medium Gaussian basis sets.^7–10^ Although their successful application to molecules and molecular crystals,^8–11^ the originally proposed methods were of limited applicability to other kind of solids and materials. Recently, the “3c” methods have then been revised and optimized for solid-state calculations by some of us. This has resulted in a set of methods referred to as “sol-3c”.^12^ They are based on global and range-separated exchange-correlation functionals for solids,^13,14^ hybridized with 25% HF exchange, namely, PBEsol0-3c and HSEsol-3c. Indeed, they show a broader applicability from covalent and ionic solids^12^ to microporous materials^15,16^ and a good scaling for application to large systems on small-to-medium computing resources.^17^

In this work, we present new composite methods for solid-state calculations based on the mGGA r^2^SCAN functional^18^ in pure and hybrid HF/DFT variants as combined with both double-ζ (sol-def2-mSVP^12^) and triple-ζ (pob-TZVP-rev2 ^19–23^ ) quality basis sets.

The r^2^SCAN is a meta-GGA functional,^18^ developed to overcome the limitations of SCAN,^24^ which has become more and more popular in quantum chemistry for several reasons, including:

- Universal applicability: Unlike some functionals, r^2^SCAN, as well as the original SCAN, is widely applicable to different systems, such as molecules, solids, and surfaces (see ref 25 and references therein). This versatility makes it a valuable tool for scientists working in various fields of chemistry and physics.
- Robustness: r^2^SCAN is known to be more robust than other meta-GGA functionals, as it reduces some common problems such as self-interaction error, charge delocalization, and overestimation in bond energy calculations.^18,26^

- Accuracy: r^2^SCAN provides accurate results for a wide range of chemical and material properties, such as bond energies, equilibrium geometries, reaction barriers, and electronic properties.^25,26^

- Computational efficiency: Even though r^2^SCAN is more complex than GGA functionals, its computational cost remains relatively low, especially because it removes the numerical problems present in the original version of the SCAN functional which required very fine integration grids.^18^ This makes it suitable for the large-scale calculations and simulations of complex systems.

Recently, Grimme et al.^27^ have also presented the same DFT functional in its pure mGGA version compose in a “3c” fashion with a tailor-made triple-ζ basis set and semiclassical corrections. Here, the new r^2^SCAN methods are specifically targeted for solid-state calculations and are labeled “sol-3c” and “pob-3c” based on the adopted basis set. These methods have been calibrated, validated, and tested by being compared with reference benchmarks for solids (i.e., the X23,^28^ DMC8,^29^ and SS20 ^30^ databases) and selected solid-state applications. These applications include the binding of layered materials; the mechanical stability, vibrational properties, and simulated IR and Raman spectra of α-quartz; the relative stability of all-silica zeolitic polymorphs; and the adsorption of small molecules on the external surfaces of solids and the inner surfaces of porous materials.

In the following section, we briefly review the theoretical framework of the “3c” methods as revised in this work. We then discuss the results of benchmarking the r^2^SCAN-based sol-3c and pob-3c composite methods, along with evidence of their computational efficiency.

## 2 Theoretical Framework

### 2.1 Composite mGGA Methods

The total energy of r^2^SCAN-based sol-3c and pob-3c composite methods is written as^31^

1 $$E_{\mathrm{tot}}^{\mathrm{sol}-3c/\mathrm{pob}-3c}=E_{\mathrm{tot}}^{\mathrm{DFT}/\mathrm{basis}}+E_{\mathrm{disp}}^{D3}+E_{\mathrm{BSSE}}^{\mathrm{gCP}}$$

*E*_tot_^DFT/basis^ denotes the total energy evaluated at the DFT level in the target basis set expansion.

Here, the adopted DFT approximation is the r^2^SCAN exchange and correlation functional in its pure or hybrid variant r^2^SCAN0 with 25% of HF exchange. As in our previous work,^12^ the double-ζ def2-mSVP basis set was used in its revised version of solids (i.e., sol-def2-mSVP). In addition, we referred to the triple-ζ quality basis set pob-TZVP-rev2 that was specifically devised for solid-state calculations by Bredow and co-workers.^19–23^ In the last years, the latter family of atomic basis set was extended to cover almost all elements of the periodic table. Therefore, it allows to design r^2^-SCAN-based composite methods with a wider applicability for solid-state calculations.

The *E* ^DFT/basis^ total energy is supplemented with a damped atom–atom two-body dispersion energy as defined in the D3 approach^32^

2 $$E_{\mathrm{disp}}^{D3\left(\mathrm{BJ}\right)}=-\frac{1}{2}\sum_{\mathrm{AB}}\sum_{n=6,8}s_{n}\frac{C_{n}^{\mathrm{AB}}}{R_{\mathrm{AB}}^{n}}f_{\mathrm{damp}}^{\left(n\right)}\left(R_{\mathrm{AB}}\right)$$

Here, *C*_*n*_^AB^ denotes the *n*th-order dispersion coefficient (orders = 6, 8) for each atom pair AB, *R* ^AB^ is their internuclear distances and *s*_*n*_ are the order-dependent scaling factors. The rational Becke-Johnson damping function^33^ has become the default in combination with D3 ^34^

3 $$f_{\mathrm{damp}}^{\left(n\right)}\left(R_{\mathrm{AB}}\right)=\frac{R_{\mathrm{AB}}^{n}}{R_{\mathrm{AB}}^{n}+{\left(a_{1}R_{0}^{\mathrm{AB}}+a_{2}\right)}^{n}}$$

The damping function incorporates radii for atomic pairs $R_{0}^{\mathrm{AB}}=\sqrt{C_{8}^{\mathrm{AB}}/C_{6}^{\mathrm{AB}}}$ and functional-specific parameters *a*_1_ and *a*_2_ that have been refitted in the present work for the different composite methods. In addition, the Axilrod–Teller–Muto^35,36^ (ATM) three-body dipole–dipole–dipole term is also included.

The removal of the basis set superposition error (BSSE) due to the use of a finite basis sets is accomplished through the geometrical counterpoise correction^37^ as the sum of atomic contributions scaled by the parameter σ

4 $$E_{\mathrm{BSSE}}^{\mathrm{gCP}}=\sigma \sum_{a}^{\mathrm{atoms}}E_{a}^{\mathrm{atom}}$$

The atomic contributions are obtained as the product of the factors *e*_a_^miss^, which measure the incompleteness of the basis set chosen for atom a, and a function *f*_dec_(*R*_ab_) that depends on the interatomic distance.

5 $$E_{a}^{\mathrm{atom}}=\sum_{b\neq a}^{\mathrm{atoms}}e_{a}^{\mathrm{miss}}f_{\mathrm{dec}}\left(R_{\mathrm{ab}}\right)$$

The *e*_a_^miss^ values are obtained from the difference in atomic energy between a large basis set close to the complete-basis set limit and a smaller basis set used as target, in the presence of a weak electric field.

6 $$e_{a}^{\mathrm{miss}}=E_{\mathrm{target}}-E_{\mathrm{larger}}$$

This construction, based on atomic contributions, makes it possible to account for both intramolecular and intermolecular BSSE corrections. To obtain these values, a weak electric field (*F* = 0.06 au) is applied; otherwise, in the atomic ground state, the polarization functions included in the basis set would remain unoccupied. The second term, *f*_dec_(*R*_ab_), accounts for the decay of the BSSE with increasing interatomic distance and has the following form

7 $$f_{\mathrm{dec}}\left(R_{\mathrm{ab}}\right)=\frac{\exp\left(-\alpha R_{\mathrm{ab}}^{\beta }\right)}{\sqrt{S_{\mathrm{ab}}N_{b}^{\mathrm{virtb}}}}$$

where α and β are parameters, *N*_*b*_^virt^ is the number of virtual orbitals for atom b, and *S*_ab_ is the overlap integral between two valence Slater-type orbitals centered on atoms a and b, which can be represented as s-type orbitals whose exponents are scaled by a fourth parameter η. The difference in atomic energy between a large (nearly complete) basis set and the target basis set for each free atom is used as a measure of the basis set incompleteness error (BSIE) to generate the repulsive potential *E*_a_^atom^ with fitting parameters α, β, η (see Section S1 of the Supporting Information for further details).

### 2.2 Parameters’ Fit

All method- and basis set-related parameters were optimized for each new r^2^SCAN-based composite method, with reference to high-level CCSD­(T)/CBS binding energies and intermolecular distances for a data set of molecular dimers. Unlike our previous work, we referred to a subset of the NENCI-2021 data set.^38^ As with S66 × 8, which was originally used to fit the sol-3c methods, the selected subset contains the same 66 dimers formed by combining 14 monomers of different sizes and functional groups, characterized by different weak and dispersive interactions. More specifically, the set comprises 23 hydrogen-bonded, 23 dispersion-dominated, and 20 mixed-interaction complexes. Its elemental coverage is restricted to H, C, N, and O. In the NENCI-2021 data set, the potential energy surface (PES) is sampled by selecting six geometries relative to the equilibrium geometry, where the intermolecular interaction vector, defined by the centers of mass of the molecules forming the adduct, is scaled to 0.7, 0.8, 0.9, 0.95, 1.05, and 1.10 times the equilibrium length. This range provides better sampling of the PES in the repulsive region than the S66 × 8 data set, for which the sampling points extend toward longer intermolecular distances. As the current composite methods target solid-state systems, where interatomic and intermolecular distances are typically shorter, the selected NENCI-2021 S66 × 7 subset enabled us to enhance the gCP correction descriptions.

The optimized parameters were then determined through a two-step process by means of a global fitting of get near-optimal values, followed by a manual refinement procedure. Greater adaptability in the combined D3/gCP scheme was obtained by jointly optimizing the two D3 coefficients (*a*_1_ and *a*_2_) together with the gCP parameters α, β, and η. Table [Table tblI] lists the final D3 and gCP parameters obtained from the fitting procedure, along with the basis set expansion and the amount of Fock exchange incorporated into the new r^2^SCAN-based composite methods. The value of σ, *s*_6_, and *s*_8_ was fixed to 1.00, 1.00, and 0.00, respectively, as originally proposed for the “3c” methods.^8,9^ Although it is not easy to find a trend in the values of the fitted parameters, it is interesting to note that the parameters of the gCP correction are clearly dependent on the target basis set, while the ones of the D3 depend on both the basis set and method, in that they change from sol-3c to pob-3c and also from r^2^SCAN to r^2^SCAN0.

**1 tblI:** Summary Features of the r^2^SCAN-Based Composite Methods

	r^2^SCANsol-3c	r^2^SCAN0sol-3c	r^2^SCANpob-3c	r^2^SCAN0pob-3c
AO basis set	sol-def2-mSVP	sol-def2-mSVP	pob-TZVP-rev2	pob-TZVP-rev2
HF exchange %	0	25	0	25
D3	yes	yes	yes	yes
a_1_	0.639	0.575	0.912	0.779
a_2_	5.947	5.742	7.630	6.934
gCP correction	yes(damped)	yes (damped)	yes (damped)	yes (damped)
η	1.331	1.349	3.293	3.318
α	0.297	0.298	0.344	0.363
β	1.952	1.956	2.362	2.313
σ	1.00	1.00	1.00	1.00

### 2.3 Computational Details

The revised composite methods for solids have been implemented in a development version of the CRYSTAL23 code.^39,40^ In the CRYSTAL code, the crystalline orbitals are represented as a linear combination of Bloch functions, with each of them being built from atom-centered atomic orbitals (AOs), which are expressed in terms of Gaussian-type functions. In contrast to plane-wave codes, the implementation of Fock exchange is easier in terms of AO and well-established in CRYSTAL for more than three decades.^41,42^ CRYSTAL is the ideal program for large-scale solid-state applications as it can employ all point- and space-group symmetries. Furthermore, it scales well on high-performance computational facilities with up to 30,000 CPUs,^43^ and a version for GPUs was recently presented.^44^

For the evaluation of the DFT exchange-correlation term, a pruned XXXLGRID (150, 1454) grid, typically used for mGGA functional, was used. Tolerances for calculating the one- and two-electron integral were set to 10^–7^ and 10^–7^ for Coulomb and to 10^–7^, 10^–7^ and 10^–25^ for the exchange series, respectively. The shrinking factor for the diagonalization of the Hamiltonian matrix in the reciprocal space was set to 8 for the Monkhorst–Pack net and to 8 for the Gilat net, except for the large-scale calculation of metal–organic frameworks where we use 2 and 2.

## 3 Results and Discussion

As the target of the new composite methods is the fast and accurate prediction of structural properties, inter- and intramolecular noncovalent interactions, and other relevant properties of solids, such as band gaps, bulk moduli, vibrational frequencies, and adsorption energies, we benchmarked the performance of the present r^2^SCAN-based composite methods to several data sets of periodic systems and typical solid-state applications as detailed below. To provide an additional reference to a commonly employed hybrid DFT method, results for selected systems were also compared against calculations performed with the PBE0 hybrid functional, including the D3 dispersion correction.

### 3.1 Molecular Adducts and Molecular Crystals

#### 3.1.1 s66 × 7 Data Set

Let us begin with the s66 × 7 subset of the NENCI-2021 data set of molecular adducts,^38^ which has been used to optimize the parameters included in the D3 and gCP corrections. As reported in Table [Table tblII], on average and not unexpectedly, all r^2^SCAN-based methods show a similar value for the MARE (%) in the prediction of the intermolecular equilibrium distance. This means that for the different variants, the best-fit procedure leads to parameters that provide more or less the same accuracy. A similar trend is observed for the binding energies. Although the previous sol-3c composite methods were fitted to the S66 × 8 data set, the statistical analysis for both the intermolecular equilibrium distance and the binding energies gives values in agreement with those reported in ref 12.

**2 tblII:** Statistical Analysis of Results for Four Benchmark Sets, Analyzed in This Work, as Obtained for r^2^SCAN-Derived Composite Methods

data set	prop.		r^2^SCANsol-3c	r^2^SCAN0sol-3c	r^2^SCANpob-3c	r^2^SCAN0pob-3c
S66 × 7	Dist.	MARE (%)	1.03	1.03	1.03	1.00
(NENCI-2021)	BE	MAE (kcal/mol)	0.49	0.49	0.48	0.48
X23	Vol.	MARE (%)	2.87	3.10	1.79	2.07
	LE	MAE (kcal/mol)	1.90	1.55	1.84	1.83
DMC8	CE	MAE (kcal/mol)	2.52	1.75	1.34	0.59
SS20	LP	MAE (Å)	0.04	0.03	0.04	0.03
	BG	MAE (eV)	1.11	0.93	1.22	1.15
	BM	MAE (GPa)	6.53	6.82	9.43	11.24

#### 3.1.2 X23 and DMC8 Data Sets

The first data set of periodic systems we will consider is the X23 set, which comprises 23 organic molecular crystals. This benchmark data set is one of the most widely used in the literature for lattice energies and volumes,^28^ especially for calibrating and validating density functional approximations. Here, we refer to the updated, back-corrected experimental data for the lattice energies and molar unit cell volumes recently presented by Boese and co-workers, known as the X23b set.^45^ The reference energies in X23b are accurate to within 1–2 kcal/mol.

Figure 1 illustrates the results obtained with our r^2^SCAN-based methods for molar volumes (top) and lattice energies (bottom), with statistical analysis provided in Table [Table tblII]. First, let us look at the unit cell volumes. A closer examination reveals good agreement between the predicted and reference unit cell volumes across the entire X23 data set. However, larger deviations are observed for polar and nonpolar van der Waals bonded molecular crystals when double-*zeta* quality basis sets are used (i.e., r^2^SCANsol-3c and r^2^SCAN0sol-3c).

**Figure 1:**
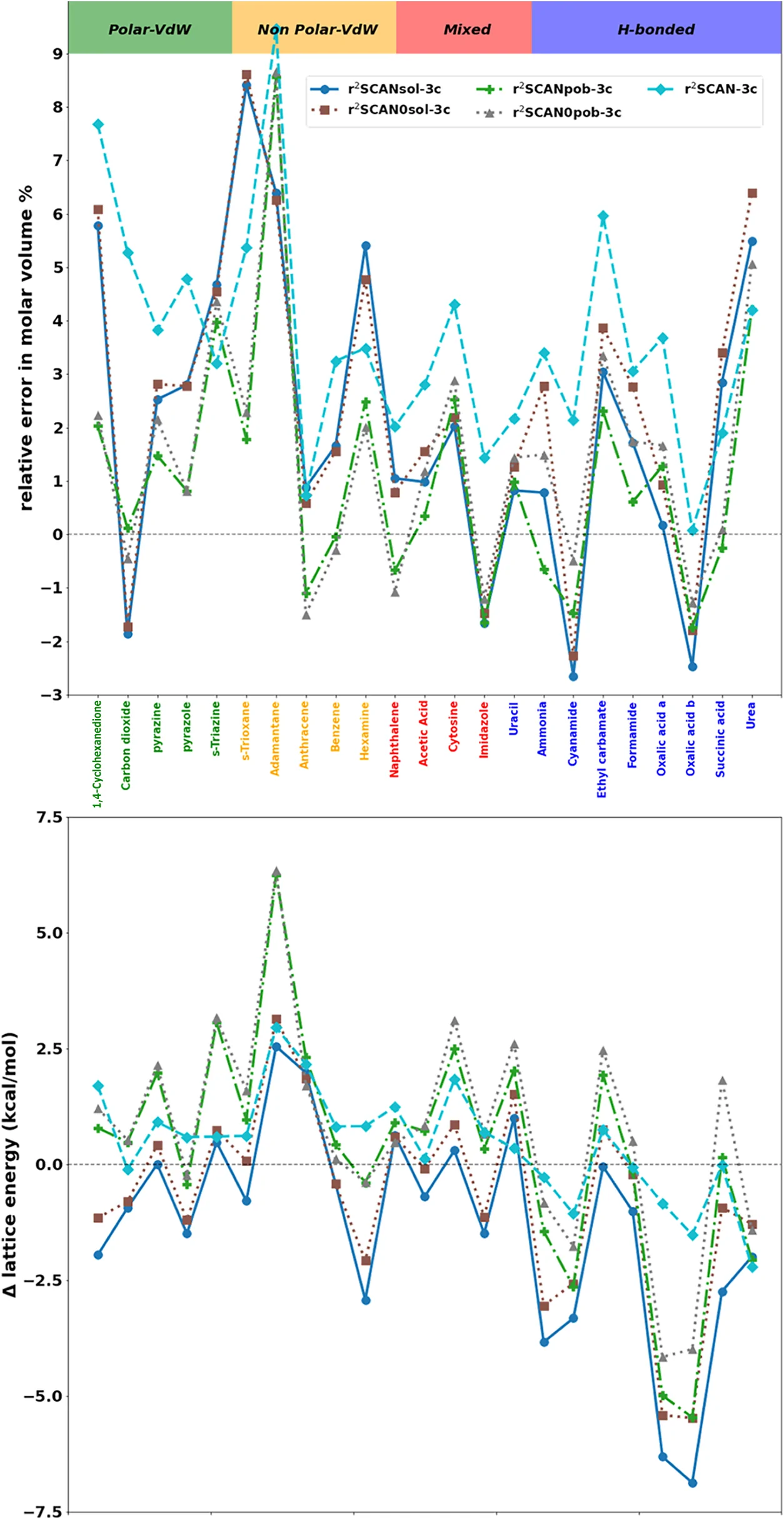
Relative error between back-corrected experimental and computed molar volumes for X23 data set. r^2^SCAN-3c data are taken from ref 27 Error between back-corrected experimental and computed unit lattice energies for the X23 data set. r^2^SCAN-3c data are taken from ref 27.

As expected, smaller errors were obtained by increasing the basis set expansion from sol-def2-mSVP to pob-TZVP-rev2. The overall accuracy of our r^2^SCAN-derived methods for unit cell volume is comparable to or better than the original “3c” methods proposed by Grimme. However, the hybrid methods perform slightly worse than their pure meta-GGA counterparts for this physical quantity, as indicated by the MARE(%) values reported in Table [Table tblII]. For lattice energies, r^2^SCAN-based composite methods perform well on the X23b data set, with all achieving MAEs below 2.0 kcal/mol; r^2^SCAN0sol-3c is the best performer, while r^2^SCANpob-3c and r^2^SCAN0pob-3c show no significant statistical difference, as indicated by their similar MAEs. The largest deviations in lattice energies are observed for adamantane, with errors exceeding 5.0 kcal/mol, and for strongly hydrogen-bonded crystals such as oxalic acid, where errors are below −5.0 kcal/mol. In the case of oxalic acid, this systematic underbinding is probably due to the absence of low-exponent basis functions, which are crucial for accurately describing such interactions. Interestingly, r^2^SCAN0sol-3c results (MAE = 1.55 kcal/mol) are of the same quality of triple-ζ DFT calculations such as B3LYP-D*/TZP, that provides an MAE of 1.15 kcal/mol. Even though they are not as accurate as extremely costly post-HF techniques such as Diffusion Monte Carlo and multilevel CCSD­(T) methods, which yield MAEs of 0.62 and 0.88 kcal/mol, respectively, it can be considered highly competitive in the trade off between cost and accuracy. To further compare the proposed composite schemes with conventional periodic DFT calculations, we considered benzene as a representative case and performed standard PBE0-D3­(ABC) calculations without the BSSE correction. Using the sol-def2-mSVP and pob-TZVP-rev2 basis sets, the calculated lattice energies are −18.87 kcal/mol and −19.87 kcal/mol, respectively, compared with the X23b reference value of −13.10 kcal/mol. Thus, conventional PBE0-D3­(ABC) calculations substantially overbind the benzene crystal, with an absolute deviations of 5.77 and 6.77 kcal/mol. Notably, the calculated BSSE of 5.81 kcal/mol for sol-def2-mSVP and 6.25 kcal/mol for pob-TZVP-rev2 is comparable in magnitude to the corresponding deviations from the reference lattice energy, suggesting that the BSSE is a major contributor to the observed overbinding. This trend is further supported by the additional calculations reported in Tables S17, S18 and Figure S1, which show that, for benzene, naphthalene, and anthracene, the magnitude of the BSSE correction increases nearly linearly with the percentage reduction in molar volume, highlighting the direct connection between basis set incompleteness, overbinding, and structural contraction. For benzene, the corresponding molar volumes are 108.56 Å^3^ and 106.17 Å^3^, respectively, compared with the reference value of 111.99 Å^3^, corresponding to relative deviations of −3.0% and −5.2%. Increasing the basis set size does not improve the agreement for this system, but instead leads to stronger binding and a larger underestimation of the volume, consistent with the substantial BSSE that persists with the pob-TZVP-rev2 basis set. Although this single case does not provide a statistically comprehensive comparison, it illustrates that a conventional hybrid-functional calculation supplemented only by a dispersion correction does not necessarily provide improved accuracy over the proposed 3c approaches.

As an additional molecular crystal data set, we examined DMC8, which contains highly accurate fixed-node diffusion Monte Carlo energies. The performance of our r^2^SCAN-based methods consistently improves as we progress from the most cost-effective r^2^SCANsol-3c to the computationally intensive r^2^SCAN0pob-3c, as evidenced by the MAE values shown in Table [Table tblII]. In particular, r^2^SCAN0pob-3c shows a remarkable performance, achieving an MAE of 0.6 kcal/mol, which is comparable to the threshold for chemical accuracy of the Grimme’s original r^2^SCAN-3c method (MAE = 0.6 kcal/mol).^27^

### 3.2 Inorganic Solids: SS20

The new r^2^SCAN-derived composite methods are expected to have wide application in solid-state calculations, extending beyond molecular crystals. We therefore assessed their accuracy in predicting the lattice constants, band gaps and bulk moduli of the SS20 data set (see ref 12), which contains 20 inorganic solids (both ionic and covalent) with cubic symmetry. Figure 2 illustrates the deviation of these calculated physical quantities from the experimental reference data cited in ref 30. As summarized in Table [Table tblII], all r^2^SCAN-based methods yield highly accurate structural results with a mean absolute error (MAE) below 0.05 Å. The only exceptions are LiF and MgO, which are systematically overestimated. For band gaps, the calculated values show an average MAE of 1.10 eV. As expected, the largest errors are observed for ionic compounds with medium to wide gaps, such as LiF, MgS and NaF. For bulk moduli, there is a slight degradation in accuracy when moving from double to triple-ζ composite methods, but this is due to the limitations of the reference data. The largest errors affect both ionic (e.g., LiF and MgS) and covalent systems (e.g., BN, diamond, GaN, Si and SiC), particularly for stiffer materials.

**Figure 2:**
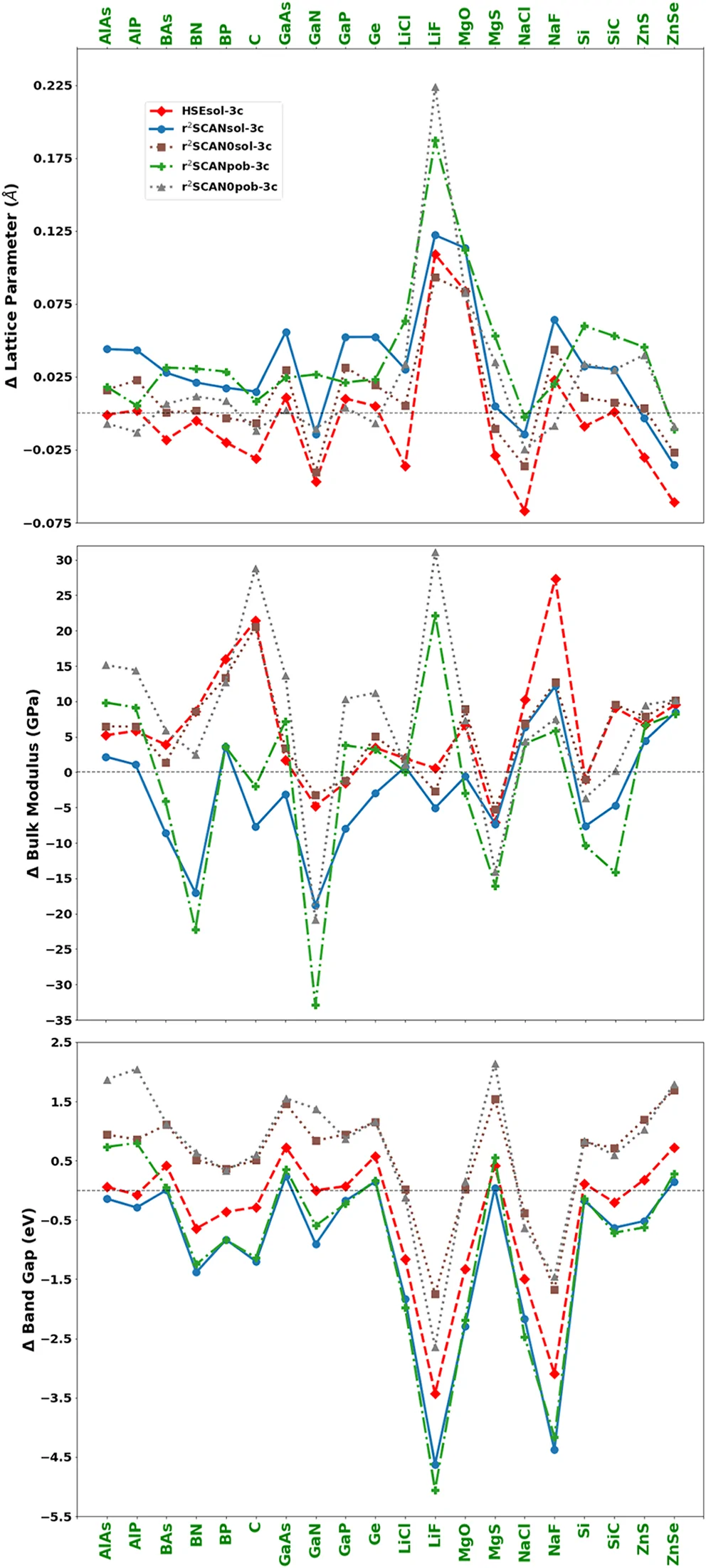
Relative error on lattice parameters, bulk modulus, and band gap for the SS20 cubic data set, computed using r^2^SCAN-derived composite methods and compared to experimental data.

The present r^2^SCAN-based methods perform similarly to our previously proposed sol-3c composite method. For comparison, Figure 2 also illustrates the results for HSEsol-3c: r^2^SCAN0sol-3c provides results that are very similar, except for the band gaps, for which the screened-Coulomb hybrid functional is more accurate. Overall, the pure r^2^SCAN composite methods predict band gaps that are not far from those of the hybrid method. This confirms that the r^2^SCAN mGGA functional improves the description of the electronic structure of solids compared to lower rungs of the Jacob’s Ladder. For the SS20 data set, the PBE0-D3­(ABC) reference results were obtained using the *sol*-def2-mSVP and pob-TZVP-rev2 basis sets. No gCP correction was applied to these calculations. The corresponding statistical errors are reported in Table S22. For the lattice constants, PBE0-D3­(ABC)/pob-TZVP-rev2 gives an MAE of 0.03 Å, compared with 0.04 Å for PBE0-D3­(ABC)/*sol*-def2-mSVP, indicating a moderate improvement upon enlargement of the basis set. In contrast, the use of the larger basis set does not improve the bulk moduli or band gaps: the corresponding MAEs increase from 8.85 to 9.74 GPa and from 0.80 to 1.03 eV, respectively. These results show that increasing the basis set size alone does not lead to a systematic improvement for all of the investigated properties. They also highlight the practical advantage of the 3c methodology, which achieves comparable accuracy for structural and mechanical properties using a more compact basis set and specifically designed corrections. In this respect, it is worthy to mention that r2SCAN-3c composite method could not be tested on the SS20 set because the molecular basis set is not suitable to solid-state calculations due to linear dependencies and SCF convergence problem.

### 3.3 Layered Materials

As a further validation test in solid-state calculations, we consider layered materials. From a computational perspective, these systems are challenging due to the combination of strong covalent bonds within layers and weak van der Waals interactions between layers. Here, we tested graphite, hexagonal boron nitride (h-BN), and black phosphorus (black P), for which high-level theoretical reference data on binding energy and low-temperature experimental data on unit cell volume are available.^46–51^ Our composite methods demonstrate excellent agreement with both available experimental data and results obtained from CCSD­(T) and QMC. However, it is important to emphasize that we found significant variability between the reference data used for comparison. This variability, which can be found in both the experimental and theoretical data reported in the literature, may be due to various factors, such as different experimental conditions (e.g., temperature, pressure, and sample purity) or the different theoretical approximations employed in other studies. This makes it difficult to make an unambiguous comparison and, in some cases, limits the possibility of definitively validating our results. Despite these difficulties, our methods have proven to be particularly robust, providing consistent predictions within the reported uncertainties of the reference data. This consistency suggests that the parametrization of the dispersion correction and the geometrical counterpoise in our methods can be considered reliable and balanced. More specifically, all r^2^SCAN-derived composite methods accurately predict the structural properties of graphite, although they systematically underestimate the interlayer binding energy.

For graphite, the PBE0-D3­(ABC)/*sol*-def2-mSVP method yields an equilibrium volume of *V* = 34.7 Å^3^, which is in reasonable agreement with the experimental data. Conversely, with the pob-TZVP-rev2 basis set, PBE0-D3­(ABC), underestimates this structural feature, with an optimized lattice volume of *V* = 33.3 Å^3^. A systematic overestimation of the binding strength is clearly observed for both methods, with values of *E*_bind_ = −58.9 meV/atom (*sol*-def2-mSVP) and *E*_bind_ = −69.5 meV/atom (pob-TZVP-rev2), respectively.

We also plotted the curves of the interlayer energy vs the lattice parameter *c* (Figure S2) to show that the CP correction for PBE0-D3­(ABC) is mandatory to reduce the overestimation and obtain better results. In this respect, the role of the gCP correction in the 3c methods is crucial to properly predicting the potential energy surface. Overall, the results depend more on the basis set rather than the adopted functional.

For h-BN, a slightly different trend emerges: only the triple-ζ methods yield accurate binding energies compared to the quantum Monte Carlo (QMC) reference data,^49^ although a clear comparison is not possible due to the wide range of values in the reference data. Similar unit cell volumes are predicted by all r^2^SCAN composite methods, as illustrated in Figure 3 (center). Analogously, a greater variability in the reference data is observed for black *P* (see Figure 3 (bottom)). Here, the unit cell volume is accurately reproduced by r^2^SCAN composites, though it is slightly underestimated by the double-ζ methods but overestimated by the triple-ζ methods. Consequently, the interlayer energy predicted by r^2^SCANsol-3c and r^2^SCAN0sol-3c is overestimated in comparison to the QMC and CCSD­(T) reference results, whereas the triple-ζ composite methods underestimate it. For these systems, using the r^2^SCAN functional leads to better performance than previous “sol-3c” methods, probably due to its ability to accurately describe regions with different types of chemical bonding.

**Figure 3:**
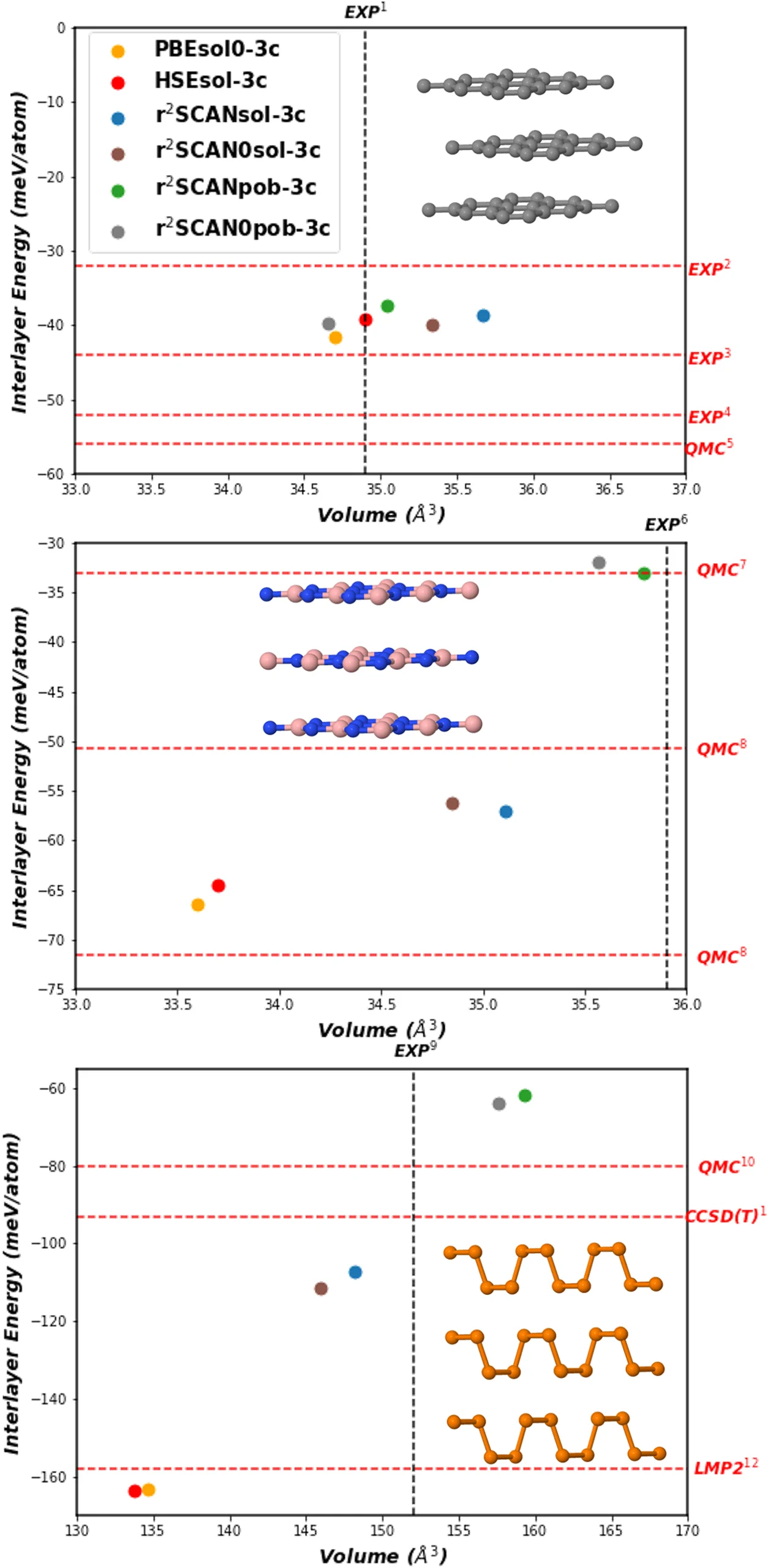
Interlayer binding energy (meV/atom) and equilibrium volume (Å^3^) for graphite (top), h-BN (middle) and black P (bottom) calculated with the r^2^SCAN-derived composite methods, compared with low-temperature structural data and high-level theoretical results for the interaction energy. Dashed lines denote the literature reference values: EXP^1^,^52^ EXP^2^,^53^ EXP^3^,^54^ EXP^4^,^55^ and QMC^5^,^56^ for graphite; EXP^6^,^48^ QMC^7^,^49^ QMC^8^,^57^ and QMC^9^,^58^ for h-BN; and QMC^10^,^59^ CCSD­(T)^11^,^60^ and LMP2^12^,^61^ for black phosphorus.

### 3.4 α-Quartz

In addition to the standard solid-state properties benchmarked and discussed above, response quantities such as vibrational frequencies, infrared (IR)/Raman spectra, and elastic, dielectric and piezoelectric properties are paramount for solids. To evaluate the accuracy of r^2^SCAN-based composite methods in predicting these properties, we use α-quartz as a reference system. Regarding the vibrational frequencies, rather than analyzing each normal mode, we refer to the predicted IR and Raman spectra in comparison with the experimental ones. IR and Raman intensities were calculated using the coupled-perturbed Hartree–Fock/Kohn–Sham (CPHF/KS) approach, as described in refs 62,63 . As the CPHF/KS approach is not currently available for mGGA functionals in CRYSTAL, we used the HSEsol-3c composite method to calculate the dipole and polarizability tensors. We then applied a Lorentzian broadening of 10 cm^–1^ to simulate both the IR and Raman spectra, as shown in Figure 4 (top and bottom, respectively). Overall, there is a remarkable agreement between the experimental and predicted spectra in both cases. In particular, all r^2^SCAN-based methods provide accurate vibrational frequencies, with an average MAE of 16 cm^–1^ (see Table S10). These methods also outperform the results obtained with the triple-ζ quality hybrid DFT functional PBEsol0/pob-TZVP, which has an MAE of 21 cm^–1^.^12^ Notably, the computational cost of the r^2^SCAN0sol-3c method is approximately twice as low as that of PBEsol0/pob-TZVP when evaluated using the larger pob-TZVP basis set.

**Figure 4:**
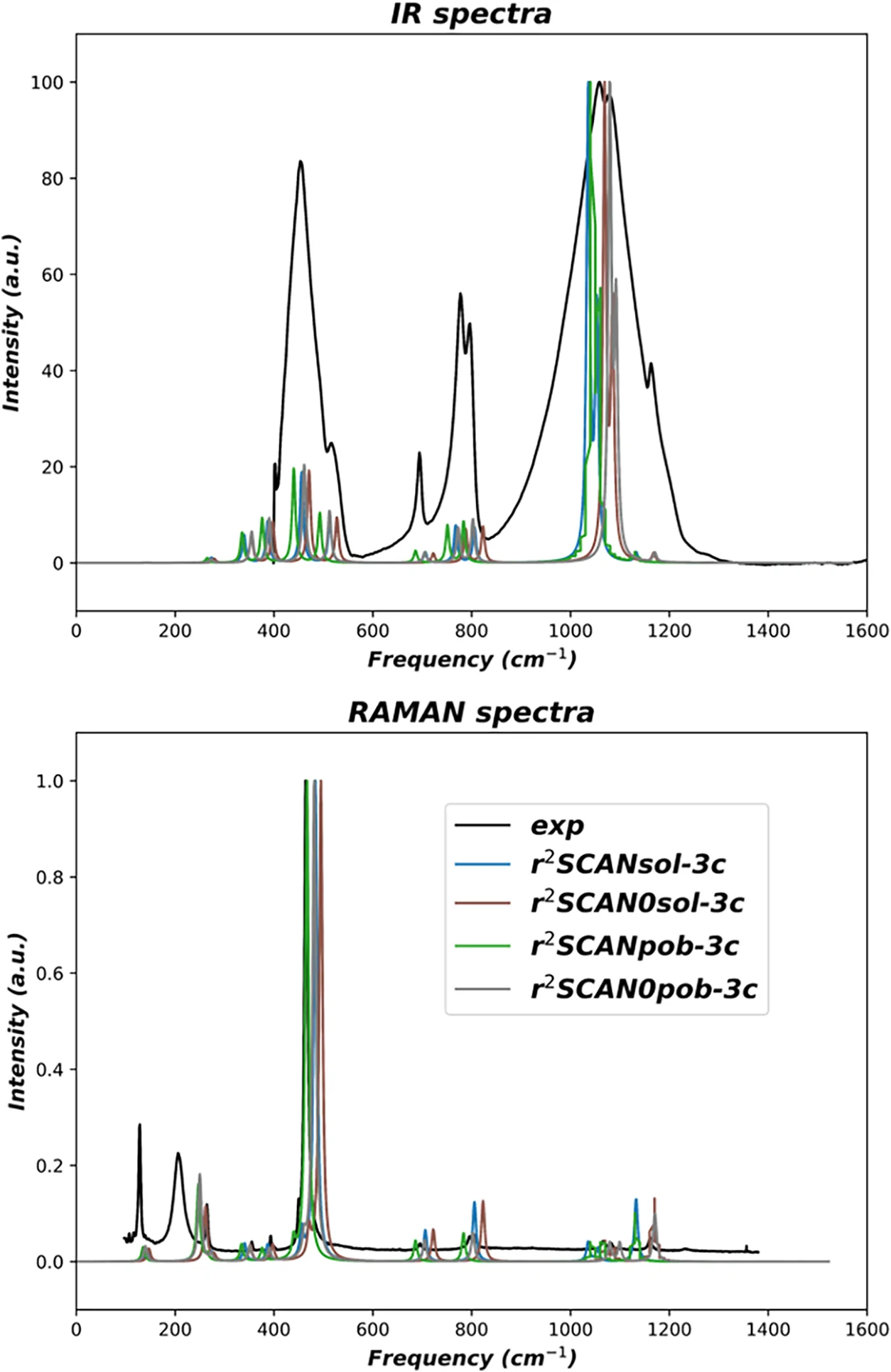
Comparison between experimental and simulated infrared (top) and Raman (bottom) spectra of α-Quartz, as computed with the r^2^SCAN-based composite methods.

α-Quartz is a material of significant technological interest due to its combination of elastic and piezoelectric properties, which are critical for applications in electronic and optical devices. Table [Table tblIII] shows the lattice parameters, second-order elastic constants *C*_*ij*_, the bulk modulus *B* and the direct piezoelectric constants *e*_*ij*_ of α-Quartz predicted with the r^2^SCAN-derived composite methods. Comparing these with experimental data shows a good agreement for the crystalline structure and most of the diagonal elastic constants such as *C*_11_, *C*_44_, and *C*_66_. However, off-diagonal terms such as *C*_12_, *C*_13_, and *C*_14_ show larger deviations. These discrepancies are expected due to the small magnitudes of these constants and are consistent with the variations observed in several experimental studies.^64–66^ All computational methods provide a stiffer description of the material than the experimental data, as indicated by a clear overestimation of the bulk modulus. The piezoelectric properties of α-quartz have been extensively measured, and there is quite good agreement between our theoretical results and the experimental findings^64,66,67^ . Comparing sol-3c and pob-3c methods shows an overall softening of the structure, which is linked to a decrease in the *C*_33_ elastic constant. Instead, the piezoelectric response depends on both basis set and the amount of HF exchange with the r^2^SCAN0pob-3c method giving more consistent results with respect to experiments.

**3 tblIII:** Predicted Lattice Parameters (in Å), Elastic Constants (in GPa), Bulk Modulus (in GPa), and Direct Piezoelectric Constants (in C/m^2^) for *α*-Quartz in Comparison with Experimental Data

	exp.	r^2^SCANsol-3c	r^2^SCAN0sol-3c	r^2^SCANpob-3c	r^2^SCAN0pob-3c
*a*	4.903	4.861	4.854	4.902	4.897
*b*	5.409	5.413	5.392	5.433	5.414
*C*_11_	87.65	90.18	91.48	87.35	90.17
*C*_12_	9.42	24.91	21.89	20.34	18.78
*C*_13_	12.8	28.23	25.32	23.44	21.00
*C*_14_	–17.83	–9.77	–11.75	–11.63	–14.78
*C*_33_	107.65	138.15	135.08	123.30	124.17
*C*_44_	59.6	59.72	61.75	58.73	61.74
*C*_66_	39.12	32.64	34.79	33.51	35.70
*B*	38.75	53.47	51.45	48.05	47.34
*e*_11_	0.149	0.218	0.217	0.19	0.112
*e*_14_	–0.057	–0.097	–0.091	–0.08	–0.07

### 3.5 Phase Transition: α-Quartz vs Stishovite

Phase transitions are important phenomena in solid-state physics and materials science. In this study, we have investigated the structural phase transition of two silica (SiO_2_) phases, namely, α-Quartz and Stishovite. This involves a change in the coordination of the Si atom from tetrahedral to octahedral. This is an interesting validation test to check if meta-GGAs and, in particular the SCAN class of functionals, are able to describe the two diverse chemical bonding environments.

As shown in Table [Table tblIV], all present r^2^SCAN-based composite methods predict α-Quartz to be the most stable phase even though its equilibrium relative stability is significantly underestimated in comparison with experimental results. However, the transition volumes are accurately reproduced. Figure 5 illustrates the zero-temperature phase transition between α-Quartz and Stishovite as determined by the change in enthalpy. The predicted transition pressure are definitively underestimated with respect to both the QMC prediction of 7.0 GPa^69^ and the experimental value of 7.46 GPa even when a finite-temperature correction is included (see Table [Table tblIV]). Also, for this phase transition, it is expected that the self-interaction error can play an important role;^70^ however, the inclusion of exact exchange in r^2^SCAN0 with both sol-3c and pob-3c variants does not significantly improve upon the pure semilocal functional.

**4 tblIV:** Energies Differences (Δ*E*_0_), Volumes (Δ*V*_0_), and Transition Pressures (P_*t*_) of SiO_2_ Calculated with the r^2^SCAN-Based Composite Methods in Comparison with Experimental Results

method	Δ*E*_0_ (eV/SiO_2_)	Δ*V*_0_ (Å^3^/SiO_2_)	*P*_*t*_ (GPa)[Table-fn tIVfn1]
r^2^SCANsol-3c	0.24	12.81	4.03
r^2^SCAN0sol-3c	0.20	13.09	3.39
r^2^SCANpob-3c	0.19	13.26	3.02
r^2^SCAN0pob-3c	0.22	13.51	3.33
Exp.	0.48–0.54	14.43	7.46

a A finite-temperature correction of 0.58 GPa has been included as adopted in ref 68.

**Figure 5:**
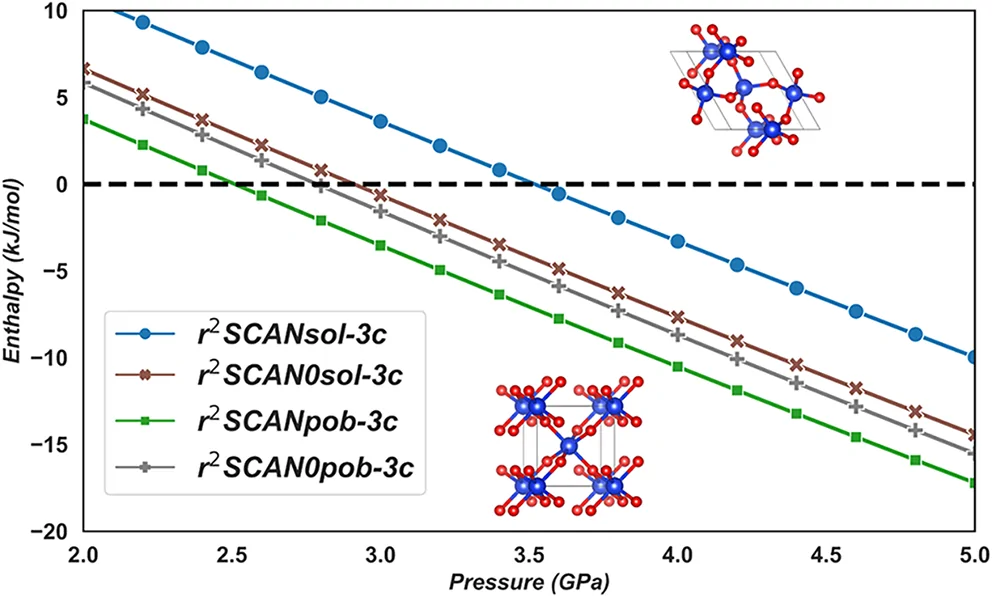
Enthalpy of the α-Quartz–Stishovite transition, as predicted with the r^2^SCAN-based composite methods without temperature correction. The stable phase of above 0.0 kJ/mol is α-quartz, while below that, stishovite is the stable phase.

As pointed out in ref 70, one possible explanation for the significant discrepancy between the predicted and experimental transition pressures is the inclusion of the dispersion correction, which has been shown to worsen the results. Another possible source of error could be the limitations of the adopted basis sets. A more reliable description of the phase transition can indeed be obtained by including a second polarization function on silicon in the POB basis set (see Table S11). For r^2^SCAN, this results in a finite-temperature corrected transition pressure of 5.11 GPa, which is closer to previous SCAN results^68,70^ (i.e., 5.2 GPa) and experimental observations.^71–73^ However, when the D3 dispersion correction is included, the transition pressure reduces to 3.95 GPa. This shows that for this kind of property, there is a delicate balance between the augmentation with a dispersion correction and the basis set quality. The dispersion correction tends to stabilize Stishovite with respect to α-quartz probably because the former is denser than the latter. For the same reason, with the present 3c methods, the gCP correction counterbalances this spurious stabilization and leads to a better agreement with the experimental data although it is still definitively underestimated (see Table S11). In particular, the transition pressure appears to be more sensitive to the overall effect of the basis set and the inclusion of D3 and gCP corrections.

### 3.6 Relative Stability of Zeolites

Polymorphism is another phenomenon of great relevance to the solid state. The existence of several different crystalline phases within a narrow energy range makes it challenging to predict their relative stabilities. In this context, the polymorphism of silica-based microporous materials, such as zeolites,^74^ provides an important validation test. Zeolites are composed of tetrahedrally coordinated silicon (Si) atoms linked together through oxygen (O) atoms. The flexibility of these Si–O–Si bridges enables various secondary building units to be created that assemble into three-dimensional porous frameworks with specific topologies. Zeolites are widely employed in catalysis, gas separation, and ion exchange processes.^75^ Consequently, accurate modeling of their properties is of great importance for practical and real-world applications.

The formation energy of zeolites has been carefully investigated by Navrotsky et al. using calorimetric measurements,^76,77^ which show that their thermodynamic stability relative to α-quartz is indeed very narrow, spanning less than 10 kJ/mol.

Figure 6 illustrates how the r^2^SCAN-based composite methods predict the relative stability of the examined set of zeolites in comparison with the corresponding back-corrected experimental data.^78^ In addition, the statistical analysis is reported in Table [Table tblV]. Among the methods presented, r^2^SCAN0pob-3c shows the highest accuracy and precision with a mean absolute error (MAE) of 7.36 kJ/mol and a standard deviation (SD) of 1.55 kJ/mol per SiO_2_ unit, which is on par with the PBEh-3c results previously reported by one of us.^78^ r^2^SCANpob-3c performs slightly worse with an MAE of 8.59 kJ/mol and an SD of 1.89 kJ/mol per SiO_2_ unit, while the double-ζ methods prove insufficient for accurate energy evaluation. Morevoer, the overall trend of the relative stability as observed experimentally is not well reproduced by the present composite methods. These discrepancies are likely due to two factors. On the one hand, as for the α-quartz vs stishovite relative stability (see above), the D3 correction gives an overstabilization of the denser α-quartz phase with respect to the porous zeolites. On the other hand, the wrong stability ranking could be due to the selected exchange and correlation functional. To better understand the role of the exchange-correlation functional, we examined the change in energy for the deformation of the Si–O–Si bridge in the simple siloxane molecule (see inset in Figure 6). With respect to the linear conformation (i.e., Si–O–Si angle of 180 deg.) smaller angles result in more stable structures. For B3LYP, the range of angles with negative Δ*E* is smaller and less stabilized than for the r^2^SCAN-based methods. This indicates that the latter tends to favor a wider range of structures, thus failing to capture the subtle energy differences that are critical to accurately predicting and differentiating zeolite geometries.

**5 tblV:** Statistical Analysis of the Predicted Formation Energy of Zeolites from *α*-Quartz, Expressed in kJ/mol per SiO_2_ Unit[Table-fn tVfn1]

method	ME	MAE	SD
r^2^SCANsol-3c	12.92	12.92	2.19
r^2^SCAN0sol-3c	11.69	11.69	1.96
r^2^SCANpob-3c	8.59	8.59	1.89
r^2^SCAN0pob-3c	7.36	7.36	1.55

a Values were calculated using composite methods derived from r^2^SCAN and compared with quasi-experimental data.

**Figure 6:**
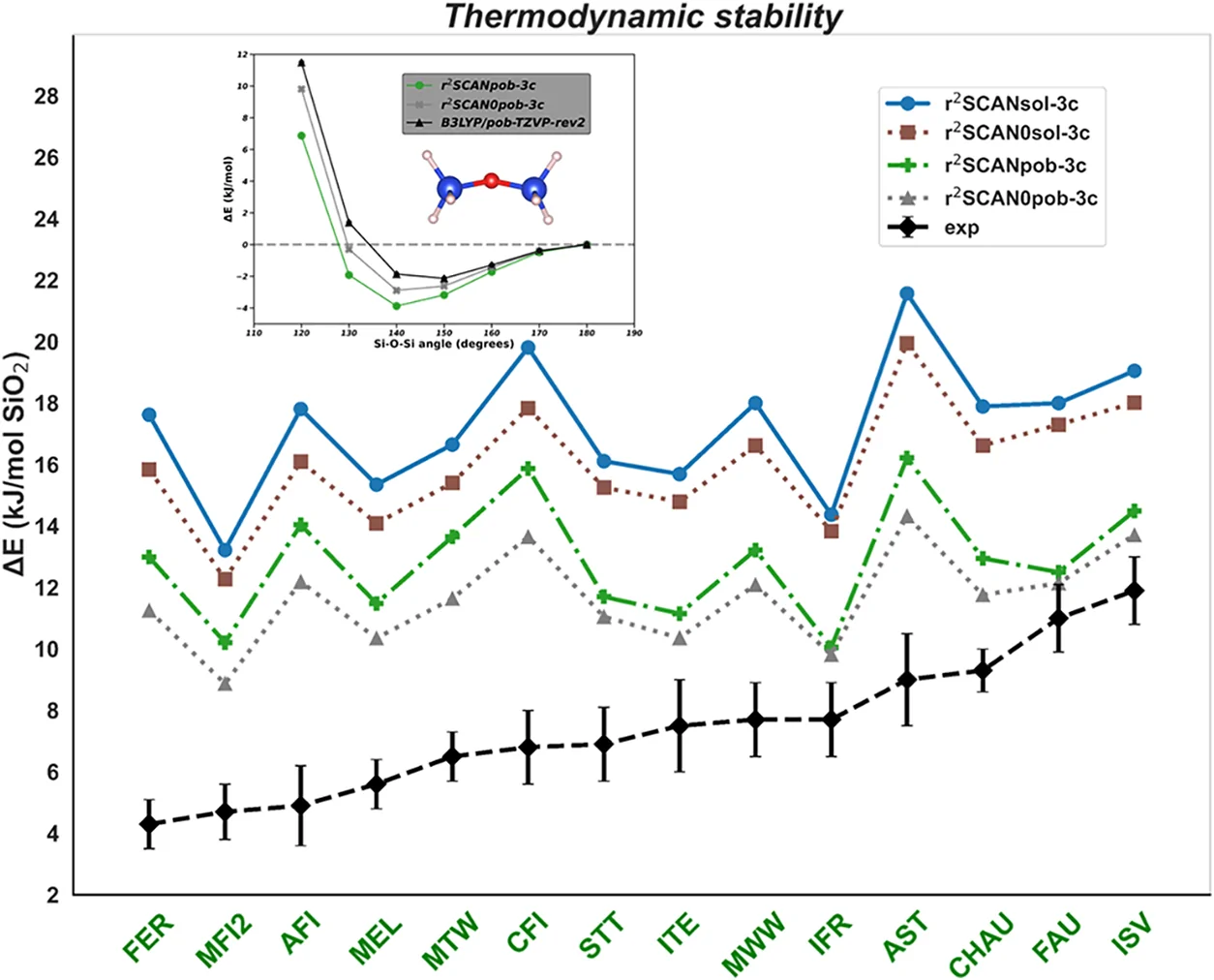
Comparison between calculated and back-corrected experimental relative stability of zeolites with respect to α-quartz (see text for details).

### 3.7 Adsorption on External and Inner Surfaces of Solids

The adsorption of small molecules on solid surfaces is of fundamental importance across a wide range of scientific and technological domains, including heterogeneous catalysis, gas purification, chemical sensing, material storage and separation, and the development of energy-related materials. Solid surfaces, whether external (as in inorganic materials) or internal (as in porous materials with high specific surface areas, such as metal–organic frameworks, MOFs), offer active sites capable of interacting with various molecules, thereby altering their chemical and physical properties.

In view of its relevance to advanced applications, we investigated the adsorption of small molecules such as CO, H_2_O, CH_4_, C_2_H_2_, and CO_2_ on the external surfaces of inorganic solids (i.e., MgO, NaCl) and within the inner surface of microporous materials (e.g., MOFs). This validation test represents a significant challenge, as it requires an accurate description of both molecular and periodic regimes.

The adsorption of CO on MgO(001) has been broadly investigated from a theoretical viewpoint;^79–85^ therefore, here we benchmarked our r^2^SCAN-based composite methods against recent high-level hybrid Coupled-Cluster (CC) and MP2 calculations^85^ predicting a binding energy of −21.2 kJ/mol, that we used as a reference in the following. r^2^SCAN methods combined with double-ζ basis set significantly overestimate the adsorption of CO with MgO, while triple-ζ-based methods (i.e., r^2^SCANpob-3c and r^2^SCAN0pob-3c) demonstrate improved performance, yielding results that are in closer agreement with those obtained from MP2:DFT-D+ΔCC calculations,^83^ as shown in Figure 7. A similar trend is observed for H_2_O and CH_4_ adsorption, although the overbinding is less pronounced. For C_2_H_2_ adsorption on NaCl surfaces, all r^2^SCAN-based methods perform almost equally with an overestimation of the binding energy. This overestimation is slightly greater for the double-ζ composite methods than for the triple-ζ ones, in both full (fc) and half (hc) coverage models.^86,87^

**Figure 7:**
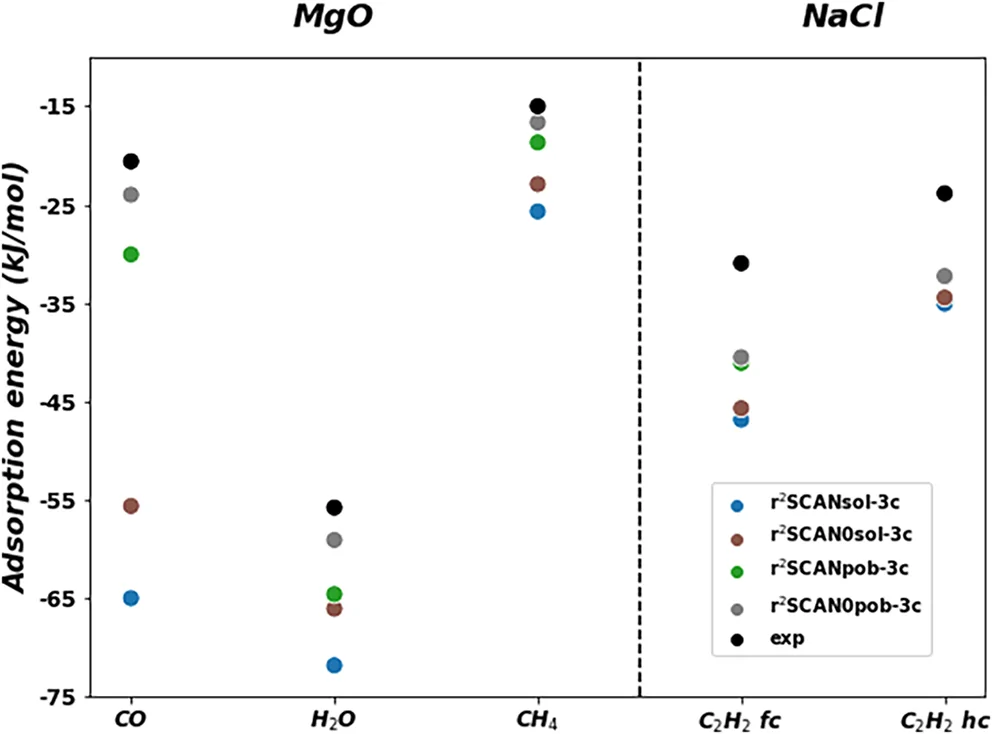
Adsorption energies (kJ/mol) of CO, H_2_O, CH_4_, C_2_H_2_, and CO_2_ on the external surfaces of MgO and NaCl as computed using the r^2^SCAN-based composite methods. Results are compared to high-quality reference data (vide infra). For C_2_H_2_ adsorption two different models namely full coverage (fc) and half coverage (hc) have been modeled.

In recent decades, porous materials such as the metal–organic frameworks have emerged as promising materials for gas separation, storage, and purification. This technological potential is due to their unique combination of high surface area and coordinatively unsaturated metal sites, which can act as Lewis acid sites and interact directly with adsorbed gas molecules.

In the following, we discuss the adsorption of small molecules, such as CO and CO_2_, on CPO-27-M (M = Mg, Zn) and HKUST-1 (Cu). The computed adsorption energies are compared to experimental heats of adsorption measured at room temperature. The former should be corrected to include the zero-point vibrational energy (ZPE) and a thermal correction to the enthalpy at the given temperature. However, this contribution has been predicted to be small, about 3.5–5.0 kJ/mol for CPO-27­(M) at room temperature;^88^ therefore, it has not been explicitly considered in the present work.

For CO, all composite methods, especially those based on the sol-def2-mSVP basis set, show a tendency to overestimate the adsorption energy between the gas molecules and the open metal sites, as testified in Figure 8. This was already mentioned in our previous work^15^ and can be attributed to the inadequacy of the double-ζ basis set in describing the CO molecule. To overcome such a problem, in the present work, we also referred to a triple-ζ basis set. Indeed, the latter improve the predicted adsorption energies and provide a better agreement with the experimental findings. In contrast, the adsorption of CO_2_ appears to be less susceptible to the basis set expansion employed by composite methods, yielding results that align reasonably well with experimental findings.

**Figure 8:**
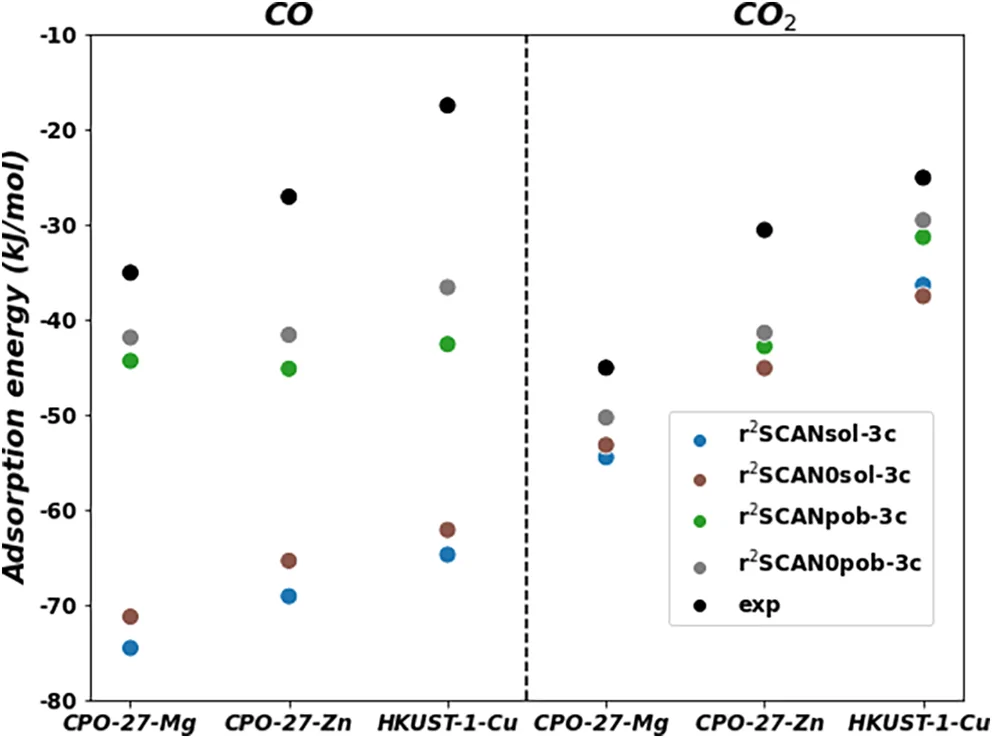
Predicted adsorption energies (kJ/mol) for CO and CO_2_ on the internal surfaces of three different MOFs containing open metal sites, computed with the r^2^SCAN-derived composite methods and compared against the experimental values.

Overall, composite methods combined with a triple-ζ basis set provide more accurate and reliable results due to the larger basis set expansion. The same holds for hybrid methods compared to their semilocal counterparts, as the inclusion of a certain amount of Hartree–Fock exchange reduces the self-interaction error, thereby mitigating the overbinding of the computed interaction energies in ionic solids and electron-rich substrates.

### 3.8 Timing: Cost-Effective Nature

To demonstrate the computational efficiency of the devised DFT composite methods, we compared the average timings for a single self-consistent cycle, on a noncovalently bound complex consisting of 148 atoms,^89^ considering the evaluation of the D3 and gCP corrections negligible. Figure 9 shows the relative timings of our new pseudohierarchy composite methods normalized to the fastest method, r^2^SCANsol-3c and compared to the r^2^SCAN-3c method proposed by Grimme.^27^ The latter approach employs a modified TZVPP basis set with 2420 basis functions (BFs), whereas the present sol-3c and pob-3c variants contain 1856 BFs and 2328 BFs, respectively. Therefore, it is important to interpret the reported timings with caution, as they significantly depend on the basis set expansion and the inclusion of exact Fock Exchange. For instance, in the selected system, the wall-clock time doubles going from a double-ζ to a triple-ζ basis set, consistently with their smaller BF expansion and with differences in basis set contraction and Gaussian exponents, which affect the numerical efficiency of the SCF procedure in the CRYSTAL^41^ implementation. Notably, the larger cost of r^2^SCAN-3c relative to r^2^SCANpob-3c cannot be explained by the number of BFs alone, since mTZVPP contains only about 4% more BFs than pob-TZVP-rev2. These differences arise from the contraction scheme and exponent distributions, and their resulting effect on integral screening and evaluation and matrix sparsity, also contribute to the observed timing difference. Meanwhile, using a hybrid functional (i.e., r^2^SCAN0) increases the computational cost by only a factor of 3 compared to its pure meta-GGA counterpart (i.e., r^2^SCAN). Overall, the proposed composite schemes demonstrate significantly faster performance than the original r^2^SCAN-3c method, along with a broader applicability.

**Figure 9:**
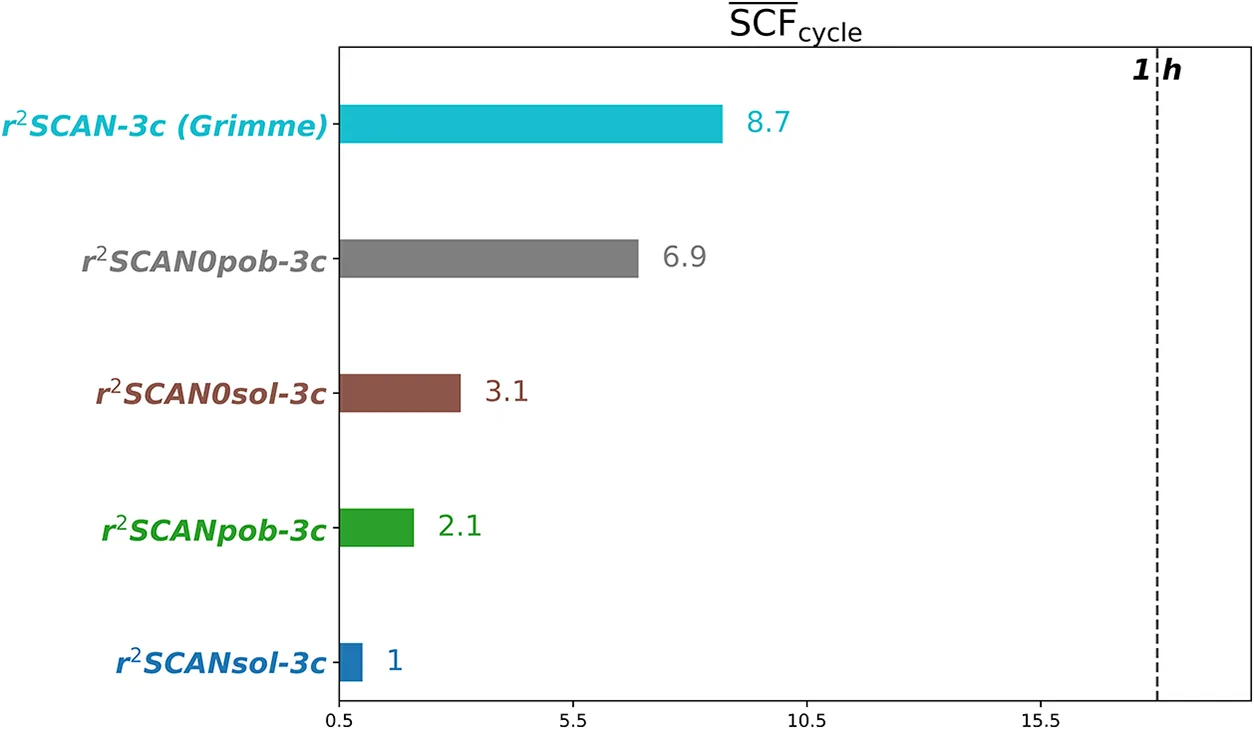
Average timings of a single SCF cycle for a noncovalently bound complex (9_AB_ from S30L,^89^ 148 atoms), evaluated using r^2^SCAN-based composite methods and compared with the r^2^SCAN-3c method developed by Grimme. Calculations were performed using Pcrystal on 16 cores of an Intel­(R) Xeon­(R) CPU E5-2650 @ 2.00 GHz.

As another example of computational efficiency, we consider the cost of calculating the interaction energy of CO_2_ in a giant-MOF such as MIL-100­(Cr). The adopted structure of MIL-100­(Cr)—with fluoride as the counterion—shows a tetragonal symmetry (space group *I*41/*amd*), which still retains 16 symmetry operators. This MOF contains 2788 atoms in the primitive cell whose number of atoms increases to 3196 when CO_2_ is put in interaction with the residual coordinatively unsaturated metal sites.^90^ Despite the large size of the system and the presence of a spin-polarized transition metal, with the r^2^SCANsol-3c method, the cost of the full SCF and the calculation of both atomic forces and stress tensor on 128 cores (AMD Epyc Turin 9535 64 core 2,4 Ghz) is about 30 min on average, thus making the full geometry optimization of the structure affordable in a couple of days without requiring high-performance computing resources.^17,90^ Interestingly, this example gives us an opportunity to point out two relevant aspects. First, it confirms that the r^2^SCAN functional improves the results for the adsorption on transition-metal sites in MOFs. Indeed, the computed interaction energy for MIL-100­(Cr)/CO_2_ is 46.8 kJ/mol in good agreement with the binding predicted with a cluster model at the CCSD­(T)/TZVP level of theory (i.e., −49.0 kJ/mol).^91^ Second, the computational efficiency of the r^2^SCANsol-3c composite method can be exploited for the most computationally demanding geometry optimization process, and then the composite method r2SCAN0pob-3c can be used for a single-point energy calculation to refine the results. For MIL-100­(Cr)/CO_2_, this approach gives an interaction energy of −44.7 kJ/mol, still in good agreement with the higher-level reference data.

Therefore, to take advantage of the cost-effectiveness of the proposed composite methods, we suggest the following general strategy. First, a geometry optimization using the r^2^SCANsol-3c composite method could be conducted and, subsequently, a single-point energy with the r2SCAN0pob-3c on the optimized structure should be computed (see Tables S1, S7–S9, S12–16).

## 4 Summary and Conclusions

In this study, we have expanded the “sol-3c” family of composite methods to include meta-GGA methods, employing the r^2^SCAN exchange-correlation functional. These methods combine r^2^SCAN or its hybrid variant, r^2^SCAN0 (25% Fock exchange), with solid-state double- or triple-ζ quality Gaussian basis sets, as well as the semiclassical D3 and gCP corrective potentials for London dispersion interactions and basis set superposition error, respectively. These new methods are known as r^2^SCANsol-3c, r^2^SCAN0sol-3c, r^2^SCANpob-3c, and r^2^SCAN0pob-3c.

We evaluated the accuracy of the new composite methods across a wide range of organic and solid-state data sets and systems, including molecular adducts, molecular crystals, inorganic solids with various types of chemical bonding, layered materials, and metal–organic frameworks. Several properties were calculated and predicted, ranging from basic structural features and energetics to electronic structure, vibrational frequencies, and mechanical and piezoelectric response.

Overall, the proposed composite schemes provide accurate results for a broad range of applications. Notably, the pob-TZVP-rev2-based methods (i.e., r^2^SCANpob-3c and r^2^SCAN0pob-3c) outperform the sol-3c methods in terms of interlayer binding and adsorption energies. This enhanced performance is attributed to the larger basis set, which offers greater flexibility and improves the description of these particular properties and chemical environments. A combined hybrid triple-ζ and semilocal double-ζ approach (i.e., r^2^SCAN0pob-3c//r^2^SCANsol-3c) was also suggested that can effectively give good structures (sol-3c) and improve the energetics (pob-3c), thus reducing the computational burden of structure relaxation in large molecular and solid-state systems.

However, limitations remain for certain applications such as SiO_2_ phase transitions and zeolite stability. These limitations appear to be related to the choice of the basis set and exchange-correlation functional, highlighting areas where further refinement may be necessary.

The reduced computational cost of r^2^SCAN-derived methods, combined with their high accuracy, makes them ideal for large-scale applications. These include high-throughput computational screening of materials, crystal structure prediction, and, more broadly, the in silico design of bespoke materials for specific applications and materials discovery. Their efficiency and reliability open up new possibilities for large-scale computational studies and material discovery. For example, work is currently underway to calculate key properties such as energies, atomic forces, stress tensors, band gaps, and Hirshfeld atomic charges for a data set comprising thousands of MOFs. This data set will be instrumental in developing a general-purpose machine learning (ML) interatomic potential tailored specifically for MOFs.

## Supplementary Material



## Data Availability

The data supporting this study are available in the Manuscript, as Supporting Information. Representative input and output files are available at 10.5281/zenodo.20051233. CRYSTAL is a software distributed under commercial license (https://www.crystal.unito.it).^39,40^

## Notes

Examined data sets: X23, including lattice energies and molar volumes; DMC8, including binding energies; and SS20, including lattice parameters, bulk moduli, and band gaps; additional details on the gCP correction; structural parameters and interlayer binding energies of layered materials; vibrational frequencies of α-quartz; additional data for the α-quartz/stishovite phase transition; relative stabilities of all-silica zeolites; adsorption energies on external and internal surfaces of solids; and further PBE0-D3­(ABC) calculations on selected X23 molecular crystals, the SS20 data set, and graphite (PDF)
