# Supplementary material for: r2SCAN-Based Composite Methods for Solid-State Calculations
Source: J Chem Theory Comput. 2026 Aug 29;22(21):11251–65. doi: 10.1021/acs.jctc.6c00852 (PMC13757750; doi:10.1021/acs.jctc.6c00852)
Supplement: Supplementary file 1 [file ct6c00852_si_001.pdf]

r^2SCAN-based composite methods for solid state calculations

Mattia Raimondo, Lorenzo Donà,* and Bartolomeo Civalleri
Dipartimento di Chimica, Università di Torino and NIS Interdepartmental Center, Via P. Giuria 7, 10125 Torino, Italy

(Dated: 15 August 2026)

Supporting Information

CONTENTS

S1. Additional details on gCP correction S2

S2. Molecular Crystals: X23 and DMC8 S2

S3. Inorganic Solids: SS20 S4

S4. Layered Materials S5

S5. α-Quartz S6

S6. Phase Transition: α-Quartz vs Stishovite S6

S7. Relative Stability of All-silica Zeolites S7

S8. Adsorption on External and Inner Surfaces of Solids S7

S9. Additional Calculations: PBE0-D3(ABC) S8
A. Additional calculations on selected systems from the X23 benchmark S8
B. SS20 S10
C. Graphite S11

References S12

* Email: lorenzo.dona@unito.it

S1. ADDITIONAL DETAILS ON GCP CORRECTION

The number of virtual orbitals for atom b is obtained by subtracting the number of electron pairs from the number of basis functions, N_b^{bf}, in the basis set used as the target.

$$N_{\mathrm{b}}^{\mathrm{virt}} = N_b^{\mathrm{bf}} - \frac{N_b^{el}}{2} \tag{S1}$$

where N_b^{el} is the total number of electrons in atom b. The overlap integral, S_{ab}, is evaluated using Slater orbitals centered on each atom. The exponents of the radial part of the Slater orbitals for the valence s, p, and d shells—the latter for transition metals—are averaged to obtain the exponent of an s-type orbital.

$$\zeta_s = \eta \zeta_{\mathrm{opt},s} \tag{S2}$$

$$\zeta_s = \eta \frac{\zeta_{\mathrm{opt},s} + \zeta_{\mathrm{opt},p}}{2} \tag{S3}$$

$$\zeta_s = \eta \frac{\zeta_{\mathrm{opt},s} + \zeta_{\mathrm{opt},p} + \zeta_{d,\mathrm{opt}}}{3} \tag{S4}$$

$$S_{ab} = \langle \zeta_s \mid \zeta_s \rangle \tag{S5}$$

The optimized Slater-orbital exponents are tabulated values taken from Herman's extension of Hückel theory, while η is the fourth parameter obtained through fitting. The summation over atoms b is truncated at 30 Å without any loss of accuracy, while reducing the computational cost.

S2. MOLECULAR CRYSTALS: X23 AND DMC8

TABLE S1: Experimental and computed unit lattice energies (kcal/mol) for X23 dataset.

	Exp.[1]	r^2SCANsol-3c	r^2SCAN0sol-3c	r^2SCANpob-3c	r^2SCAN0pob-3c	r^2SCAN0pob-3c//r^2SCANsol-3c
1,4-cyclohexanedione	-21.5	-23.5	-22.7	-20.7	-20.3	-20.3
Acetic Acid	-17.6	-18.3	-17.7	-16.9	-16.8	-16.7
Adamantane	-17.2	-14.6	-14.0	-10.9	-10.8	-10.8
Ammonia	-9.2	-13.1	-12.3	-10.7	-10.1	-10.1
Anthracene	-26.4	-24.4	-24.5	-24.1	-24.7	-24.7
Benzene	-13.1	-13.5	-13.5	-12.7	-13.0	-13.0
CO_2	-7.0	-8.0	-7.8	-6.6	-6.5	-6.5
Cyanamide	-19.5	-22.8	-22.1	-22.1	-21.2	-21.2
Cytosine	-39.1	-38.8	-38.2	-36.6	-36.0	-36.1
Ethyl carbamate	-21.1	-21.1	-20.3	-19.2	-18.6	-18.7
Formamide	-19.4	-20.4	-19.6	-19.5	-18.9	-18.8
Hexamine	-20.1	-23.0	-22.2	-20.5	-20.5	-20.6
Imidazole	-21.6	-23.1	-22.7	-21.3	-20.9	-21.0
Naphthalene	-19.4	-18.8	-18.8	-18.5	-19.0	-19.0
Oxalic Acid α	-23.6	-29.9	-29.0	-28.6	-27.8	-27.5
Oxalic Acid β	-23.1	-30.0	-28.6	-28.6	-27.1	-27.1
Pyrazine	-15.4	-15.4	-15.0	-13.4	-13.2	-13.3
Pyrazole	-18.8	-20.3	-20.0	-19.3	-19.1	-19.2
s-Triazine	-15.0	-14.5	-14.2	-11.9	-11.8	-11.8
s-Trioxane	-15.4	-16.2	-15.4	-14.5	-13.8	-13.7
Succinic Acid	-31.1	-33.8	-32.0	-30.9	-29.3	-29.3
Uracil	-32.6	-31.6	-31.0	-30.5	-29.9	-29.9
Urea	-24.4	-26.4	-25.7	-26.5	-25.8	-25.8

TABLE S2: Back-corrected experimental and computed molar volumes (Å^3) for X23 dataset.

	Exp.[1]	r^{2}SCANsol-3c	r^{2}SCAN0sol-3c	r^{2}SCANpob-3c	r^{2}SCAN0pob-3c
1,4-cyclohexanedione	132.30	139.96	140.36	134.99	135.26
Acetic Acid	72.56	73.28	73.70	72.82	73.42
Adamantane	181.45	193.04	192.82	196.98	197.17
Ammonia	30.59	30.83	31.44	30.39	31.05
Anthracene	221.55	223.50	222.85	219.11	218.24
Benzene	111.99	113.84	113.73	111.94	111.66
CO_2	41.48	40.71	40.76	41.52	41.29
Cyanamide	51.04	49.69	49.88	50.29	50.79
Cytosine	111.88	114.14	114.33	114.70	115.11
Ethyl carbamate	116.80	120.36	121.32	119.50	120.71
Formamide	53.56	54.48	55.05	53.89	54.49
Hexamine	161.20	169.93	168.89	165.22	164.44
Imidazole	84.45	83.04	83.21	83.07	83.43
Naphthalene	165.48	167.21	166.78	164.37	163.70
Oxalic Acid α	74.06	74.19	74.75	75.01	75.30
Oxalic Acid β	75.65	73.78	74.29	74.34	74.69
Pyrazine	95.98	98.40	98.67	97.39	98.04
Pyrazole	83.52	85.86	85.84	84.20	84.20
s-Triazine	87.99	92.10	91.99	91.49	91.83
s-Trioxane	97.58	105.79	105.99	99.33	99.82
Succinic Acid	116.80	120.13	120.78	116.50	116.91
Uracil	111.61	112.53	113.03	112.72	113.22
Urea	70.63	74.51	75.14	73.60	74.20

TABLE S3: Experimental and computed unit lattice energies (kcal/mol) for DMC8 dataset.

	Exp.[2]	r^{2}SCANsol-3c	r^{2}SCAN0sol-3c	r^{2}SCANpob-3c	r^{2}SCAN0pob-3c
Ammonia	-8.90	-12.97	-12.20	-10.48	-9.94
Anthracene	-25.20	-24.14	-24.21	-23.69	-24.29
Benzene	-12.70	-13.25	-13.23	-12.37	-12.69
CO_2	-6.70	-7.74	-7.61	-6.48	-6.40
Ice Ih	-14.20	-18.57	-16.86	-15.81	-14.45
Ice II	-14.10	-18.56	-16.85	-16.15	-14.72
Ice VIII	-13.70	-18.21	-16.44	-16.49	-15.07
Naphthalene	-18.80	-18.66	-18.65	-18.17	-18.59

S3. INORGANIC SOLIDS: SS20

TABLE S4: Experimental and predicted Lattice Parameters (Å) for the SS20 cubic dataset

	Exp.[3]	r^2SCANsol-3c	r^2SCAN0sol-3c	r^2SCANpob-3c	r^2SCAN0pob-3c
AlAs	5.649	5.693	5.665	5.667	5.642
AlP	5.450	5.493	5.473	5.455	5.437
BAs	4.764	4.792	4.765	4.795	4.770
BN	3.592	3.613	3.594	3.623	3.603
BP	4.527	4.544	4.524	4.556	4.536
C	3.555	3.570	3.548	3.563	3.543
GaAs	5.641	5.697	5.671	5.665	5.643
GaN	4.523	4.509	4.483	4.550	4.512
GaP	5.441	5.493	5.473	5.462	5.445
Ge	5.644	5.696	5.663	5.667	5.637
LiCl	5.072	5.102	5.077	5.136	5.106
LiF	3.974	4.096	4.067	4.161	4.198
MgO	4.118	4.231	4.201	4.230	4.201
MgS	5.612	5.617	5.601	5.665	5.647
NaCl	5.565	5.551	5.529	5.563	5.541
NaF	4.570	4.634	4.614	4.590	4.562
Si	5.422	5.454	5.433	5.482	5.456
SiC	4.348	4.378	4.355	4.401	4.378
ZnS	5.399	5.396	5.402	5.444	5.439
ZnSe	5.658	5.623	5.631	5.647	5.649

TABLE S5: Experimental and predicted Bulk modulus (GPa) for the SS20 cubic dataset

	Exp.[3]	r^2SCANsol-3c	r^2SCAN0sol-3c	r^2SCANpob-3c	r^2SCAN0pob-3c
AlAs	75.0	77.2	81.5	84.8	90.2
AlP	87.4	88.5	93.8	96.6	101.8
BAs	151.1	142.5	152.5	147.0	157.1
BN	410.2	393.2	418.8	388.0	412.7
BP	168.0	171.6	181.3	171.7	180.7
C	455.9	448.2	476.4	453.9	484.7
GaAs	78.0	74.9	81.4	85.2	91.7
GaN	213.7	194.9	210.5	180.8	192.8
GaP	92.3	84.3	91.2	96.1	102.7
Ge	79.4	76.4	84.5	82.6	90.6
LiCl	38.2	38.9	40.1	38.3	39.3
LiF	76.3	71.3	73.6	98.4	107.4
MgO	169.8	169.2	178.7	166.9	177.2
MgS	78.9	71.6	73.7	62.9	64.8
NaCl	27.6	34.0	34.5	31.7	31.9
NaF	53.1	65.3	65.8	58.9	60.7
Si	101.3	93.7	100.3	90.9	97.6
SiC	229.1	224.4	238.6	214.9	229.3
ZnS	75.0	79.5	82.8	81.6	84.5
ZnSe	65.9	74.4	76.1	74.1	76.1

TABLE S6: Experimental and predicted Band Gap (eV) for the SS20 cubic dataset

	Exp.[3]	r^{2}SCANsol-3c	r^{2}SCAN0sol-3c	r^{2}SCANpob-3c	r^{2}SCAN0pob-3c
AlAs	2.23	2.09	3.17	2.96	4.10
AlP	2.49	2.20	3.35	3.29	4.54
BAs	1.46	1.47	2.57	1.51	2.59
BN	6.36	4.98	6.88	5.11	6.99
BP	2.42	1.59	2.79	1.58	2.75
C	5.49	4.29	6.00	4.35	6.09
GaAs	1.52	1.76	2.98	1.88	3.07
GaN	3.30	2.39	4.14	2.71	4.68
GaP	2.35	2.17	3.30	2.13	3.22
Ge	0.74	0.89	1.89	0.91	1.91
LiCl	9.40	7.57	9.42	7.42	9.28
LiF	14.20	9.58	12.45	9.14	11.56
MgO	7.80	5.50	7.82	5.61	7.95
MgS	5.40	5.44	6.95	5.96	7.55
NaCl	8.97	6.80	8.58	6.50	8.34
NaF	11.50	7.12	9.83	7.34	10.05
Si	1.17	1.00	1.98	1.03	2.01
SiC	2.42	1.79	3.14	1.71	3.02
ZnS	3.84	3.32	5.03	3.21	4.87
ZnSe	2.82	2.97	4.51	3.10	4.62

S4. LAYERED MATERIALS

TABLE S7: Experimental and predicted structural parameters and interlayer binding energy for Graphite, lattice parametes in Å, volume in Å^3 ed interlayer binding energy in meV/atom

	a	c	V	binding
Ref.[4,5]	2.458	6.700	34.900	-56.0
r^{2}SCANsol-3c	2.464	6.783	35.670	-38.7
r^{2}SCAN0sol-3c	2.452	6.790	35.342	-40.0
r^{2}SCANpob-3c	2.456	6.705	35.040	-37.4
r^{2}SCAN0pob-3c	2.445	6.697	34.664	-39.9
r^{2}SCAN0pob3c//r^{2}SCANsol3c				-40.1
PBE0-D3(ABC)/sol-def2-mSVP	2.455	6.642	34.700	-58.9
PBE0-D3(ABC)/pob-TZVP-rev2	2.449	6.417	33.300	-69.5

TABLE S8: Experimental and predicted structural parameters and interlayer binding energy for h-BN, lattice parametes in Å, volume in Å^3 ed interlayer binding energy in meV/atom

	a	c	V	binding
Ref.[6,7]	2.502	6.661	36.100	-50.7/-71.6
Ref.[8]	2.506	6.603	35.904	-33.2
r^{2}SCANsol-3c	2.505	6.459	35.108	-57.1
r^{2}SCAN0sol-3c	2.494	6.471	34.854	-56.3
r^{2}SCANpob-3c	2.506	6.583	35.792	-33.0
r^{2}SCAN0pob-3c	2.495	6.600	35.568	-32.0
r^{2}SCAN0pob3c//r^{2}SCANsol3c				-31.9

TABLE S9: Experimental and predicted structural parameters and interlayer binding energy for black P, lattice parametes in Å, volume in $Å^3$ ed interlayer binding energy in meV/atom

	a	*b*	*c*	*V*	binding
p-LMP2/TZVPP[9,10]	3.310	10.480	4.380	152.000	-158.0
LdrCCD[11] + ΔLCCSD(T)	3.310	10.480	4.380	152.000	-92.0
QMC[11]	3.310	10.480	4.380	152.000	-80.0
r^2SCANsol-3c	3.342	10.261	4.322	148.217	-107.3
r^2SCAN0sol-3c	3.324	10.177	4.314	145.958	-111.4
r^2SCANpob-3c	3.334	10.638	4.492	159.300	-61.7
r^2SCAN0pob-3c	3.320	10.607	4.476	157.608	-64.0
r^2SCAN0pob3c//r^2SCANsol3c					-52.2

S5. α-QUARTZ

TABLE S10: Experimental and computed vibrational frequencies for α-Quartz (TO), values in cm^{-1}.

Mode	Exp.[12]	r^2SCANsol-3c	r^2SCAN0sol-3c	r^2SCANpob-3c	r^2SCAN0pob-3c
A_1	219.0	261.2	261.6	247.0	250.0
	358.0	340.8	352.5	334.5	351.2
	469.0	483.9	494.9	466.7	481.6
	1082.0	1054.9	1086.2	1064.0	1099.4
A_2	361.3	339.6	354.4	335.6	355.0
	499.0	513.0	527.4	492.8	512.5
	778.0	767.7	788.1	751.0	772.7
	1072.0	1052.9	1084.8	1056.0	1092.0
E	133.0	144.3	147.0	135.4	139.7
	269.0	273.4	279.0	265.1	272.8
	393.5	387.4	397.7	376.2	390.6
	452.5	456.1	471.0	440.2	461.2
	698.0	706.1	722.3	686.4	705.0
	799.0	805.6	823.0	783.5	802.6
	1066.0	1035.6	1068.8	1042.2	1079.4
	1158.0	1132.5	1168.2	1130.1	1170.5
ME		-3.3	13.7	-12.5	8.1
MAE		16.4	15.3	16.3	10.7
St. Dev.		19.8	13.1	14.2	10.4

S6. PHASE TRANSITION: α-QUARTZ VS STISHOVITE

TABLE S11: Energies differences (ΔE_0), volumes (ΔV_0) and transition pressures (Pt) of SiO2 calculated with r^2SCAN-based methods in comparison with experimental results.

Method	ΔE_0 (eV/SiO_2)	ΔV_0 ($Å^3$/SiO_2)	P_t (GPa)†
r^2SCAN/POB(Si-2P)	0.33	13.28	5.11
r^2SCAN-D3(ABC)/POB(Si-2P)	0.25	12.81	3.95
r^2SCAN0/POB(Si-2P)	0.32	13.56	4.80
r^2SCAN0-D3(ABC)/POB(Si-2P)	0.21	12.97	3.31
r^2SCAN-gCP/POB	0.18	13.29	2.95
r^2SCAN/POB	6.3E-4	13.70	0.54
Exp.[13]	0.48–0.54	14.43	7.46

†: A finite-temperature correction of 0.58 GPa has been included as adopted in ref.[13]

S7. RELATIVE STABILITY OF ALL-SILICA ZEOLITES

TABLE S12: Back-corrected experimental and calculated relative stability (kJ/mol SiO_2) of all-silica zeolites with respect to α-Quartz.

Zeolite	Exp.[14]	r^2SCANsol-3c	r^2SCAN0sol-3c	r^2SCANpob-3c	r^2SCAN0pob-3c	r^2SCAN0pob-3c//r^2SCANsol-3c
FER	4.3	17.62	15.84	12.99	11.25	11.23
MFI2	4.7	13.22	12.27	10.21	8.87	
AFI	4.9	17.81	16.10	14.04	12.19	12.10
MEL	5.6	15.35	14.09	11.48	10.35	
MTW	6.5	16.65	15.40	13.65	11.64	
CFI	6.8	19.81	17.84	15.87	13.65	13.51
STT	6.9	16.11	15.25	11.70	11.05	
ITE	7.5	15.69	14.79	11.15	10.36	
MWW	7.7	18.00	16.62	13.22	12.09	
IFR	7.7	14.37	13.84	10.05	9.81	
AST	9.0	21.57	19.95	16.22	14.33	14.04
CHAU	9.3	17.89	16.62	12.95	11.76	11.51
FAU	11.0	18.00	17.30	12.50	12.14	12.02
ISV	11.9	19.05	18.01	14.49	13.71	13.55

S8. ADSORPTION ON EXTERNAL AND INNER SURFACES OF SOLIDS

TABLE S13: Experimental and predicted adsorption energies (kJ/mol) for H_2O, CH_4 and CO on the external surfaces of MgO.

	H_2O	CH_4	CO
r^2SCANsol-3c	-71.8	-25.7	-65.0
r^2SCAN0sol-3c	-66.1	-22.9	-55.6
r^2SCANpob-3c	-64.6	-18.7	-30.1
r^2SCAN0pob-3c	-59.1	-16.6	-24.0
r^2SCAN0pob-3c//r^2SCANsol-3c	-57.5	-15.8	-20.6
Exp.[15–20]	-55.8	-15.0	-20.6

TABLE S14: Experimental and predicted adsorption energies (kJ/mol) for C_2H_2 (full coverage (fc) and half coverage (hc) on the external surfaces of NaCl.

	C_2H_2	
	fc	hc
r^2SCANsol-3c	-46.9	-35.1
r^2SCAN0sol-3c	-45.7	-34.5
r^2SCANpob-3c	-41.1	-32.3
r^2SCAN0pob-3c	-40.5	-32.3
r^2SCAN0pob-3c//r^2SCANsol-3c	-40.6	-31.9
Exp.[21,22]	-31.0	-23.9

TABLE S15: Experimental and predicted adsorption energies (kJ/mol) for CO on the internal surfaces of three different MOFs containing open metal sites.

	CPO-27-Mg	CPO-27-Zn	HKUST-Cu-1
r^2SCANsol-3c	-74.5	-69.0	-64.7
r^2SCAN0sol-3c	-71.2	-65.3	-62.1
r^2SCANpob-3c	-44.3	-45.1	-42.5
r^2SCAN0pob-3c	-41.8	-41.5	-36.5
r^2SCAN0pob-3c//r^2SCANsol-3c	-40.7	-40.0	-31.0
Exp.[23]	-35.0	-27.0	-17.4

TABLE S16: Experimental and predicted adsorption energies (kJ/mol) for CO_2 on the internal surfaces of three different MOFs containing open metal sites.

	CPO-27-Mg	CPO-27-Zn	HKUST-Cu-1
r^2SCANsol-3c	-54.4	-45.0	-36.3
r^2SCAN0sol-3c	-53.1	-45.0	-37.5
r^2SCANpob-3c	-50.2	-42.7	-31.2
r^2SCAN0pob-3c	-50.2	-41.3	-29.5
r^2SCAN0pob-3c//r^2SCANsol-3c	-50.1	-41.8	-26.5
Exp.[23]	-45.0	-30.5	-25.0

S9. ADDITIONAL CALCULATIONS: PBE0-D3(ABC)

A. Additional calculations on selected systems from the X23 benchmark

In this section, we report additional calculations for selected molecular crystals belonging to the X23 benchmark set, namely benzene, naphthalene, and anthracene.

TABLE S17: Experimental and calculated molar volumes and lattice energies.

	Molar Volume (Å^3/mol)			Lattice Energy (kcal/mol)		
Compound	Exp.[1]	PBE0-D3(ABC)/sol-def2-mSVP	Reduction (%)	Exp.[1]	PBE0-D3(ABC)/sol-def2-mSVP	BSSE (kcal/mol)
Benzene	111.99	108.56	3.1	-13.1	-18.9	7.2
Naphthalene	165.48	159.73	3.5	-19.4	-26.5	10.4
Anthracene	221.55	213.46	3.7	-26.4	-34.8	13.2

TABLE S18: Experimental and calculated molar volumes and lattice energies.

	Molar Volume (Å^3/mol)			Lattice Energy (kcal/mol)		
Compound	Exp.[1]	PBE0-D3(ABC)/pob-TZVP-rev2	Reduction (%)	Exp.[1]	PBE0-D3(ABC)/pob-TZVP-rev2	BSSE (kcal/mol)
Benzene	111.99	106.17	5.2	-13.1	-19.9	7.2
Naphthalene	165.48	156.07	5.7	-19.4	-29.4	11.1
Anthracene	221.55	207.89	6.2	-26.4	-38.8	14.2

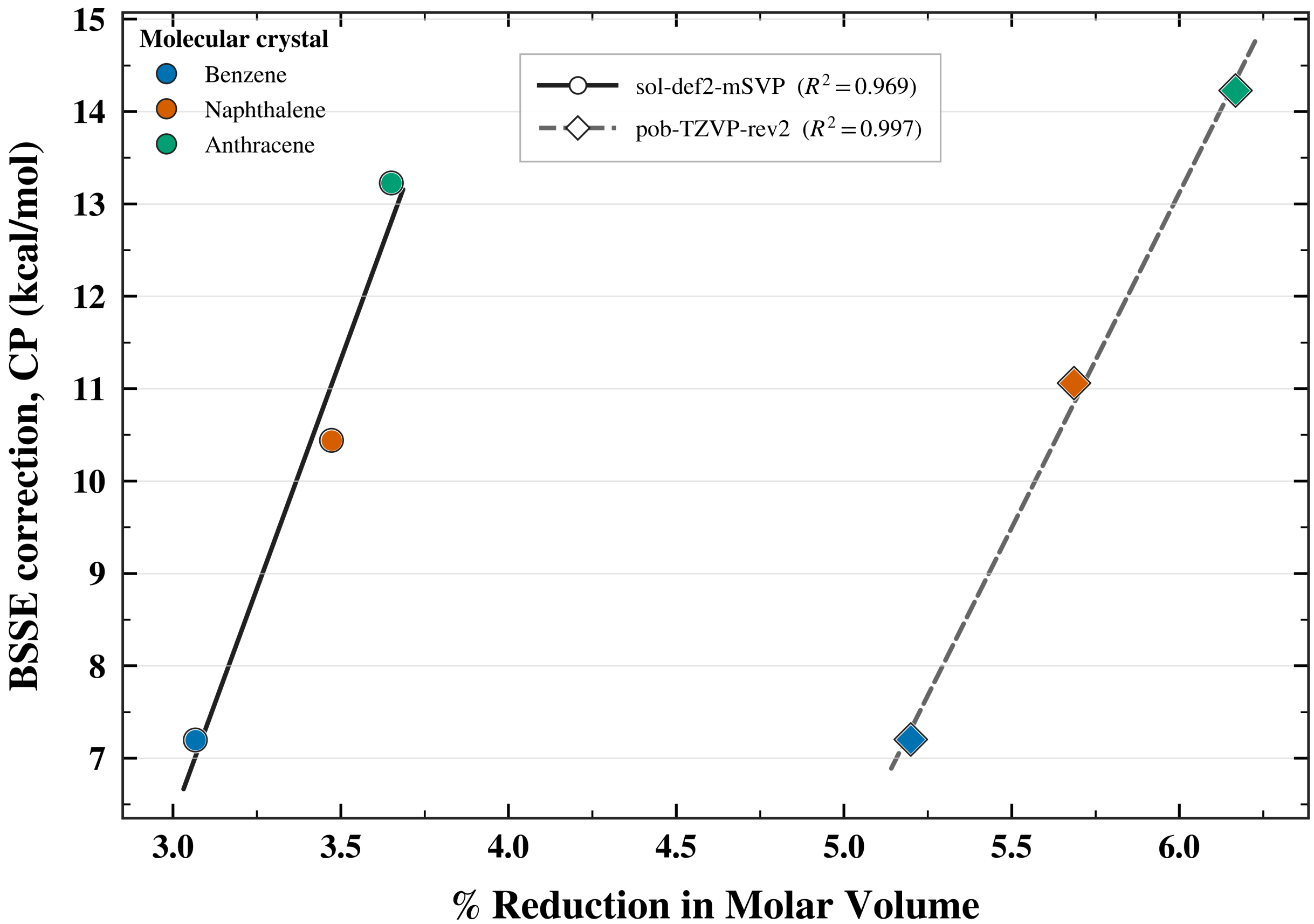

FIG. S1: Relationship between the percentage reduction in molar volume and the basis-set superposition error (BSSE) correction for the molecular crystals benzene, naphthalene, and anthracene, calculated at the PBE0-D3(ABC) level using the sol-def2-mSVP and pob-TZVP-rev2 basis sets. The lines represent linear regressions, with the corresponding coefficients of determination reported in the legend

B. SS20

TABLE S19: Experimental and predicted Lattice Parameters (Å) for the SS20 cubic dataset

	Exp.[3]	sol-def2-mSVP	pob-TZVP-rev2
AlAs	5.649	5.634	5.613
AlP	5.450	5.436	5.403
BAs	4.764	4.736	4.735
BN	3.592	3.579	3.587
BP	4.527	4.496	4.502
C	3.555	3.540	3.535
GaAs	5.641	5.650	5.612
GaN	4.523	4.480	4.492
GaP	5.441	5.447	5.413
Ge	5.644	5.496	5.617
LiCl	5.072	4.967	5.028
LiF	3.974	3.934	3.979
MgO	4.188	4.168	4.179
MgS	5.612	5.566	5.602
NaCl	5.565	5.480	5.497
NaF	4.570	4.539	4.573
Si	5.422	5.399	5.422
SiC	4.348	4.342	4.357
ZnS	5.399	5.371	5.394
ZnSe	5.658	5.598	5.599

TABLE S20: Experimental and predicted Bulk modulus (GPa) for the SS20 cubic dataset

	Exp.[3]	sol-def2-mSVP	pob-TZVP-rev2
AlAs	75.0	82.4	89.8
AlP	87.4	95.9	102.5
BAs	151.1	155.9	161.9
BN	410.2	420.4	410.1
BP	168.0	185.7	185.3
C	455.9	475.5	482.6
GaAs	78.0	81.1	91.0
GaN	213.7	206.7	208.3
GaP	92.3	92.7	102.9
Ge	79.4	82.6	88.6
LiCl	38.2	42.7	40.8
LiF	76.3	90.2	80.7
MgO	169.8	185.1	176.8
MgS	78.9	73.8	66.7
NaCl	27.6	34.0	32.3
NaF	53.1	72.8	62.5
Si	101.3	100.8	96.2
SiC	229.1	239.1	233.4
ZnS	75.0	83.6	86.0
ZnSe	65.9	77.1	76.8

TABLE S21: Experimental and predicted Band Gap (eV) for the SS20 cubic dataset

	Exp.[3]	sol-def2-mSVP	pob-TZVP-rev2
AlAs	2.23	2.95	3.86
AlP	2.49	3.12	4.29
BAs	1.46	2.48	2.49
BN	6.36	6.55	6.69
BP	2.42	2.70	2.66
C	5.49	6.01	6.06
GaAs	1.52	2.82	2.92
GaN	3.30	3.82	4.38
GaP	2.35	3.13	3.05
Ge	0.74	1.81	1.82
LiCl	9.40	9.29	9.02
LiF	14.20	12.81	12.37
MgO	7.80	7.44	7.51
MgS	5.40	6.63	7.14
NaCl	8.97	8.47	7.97
NaF	11.50	10.18	9.46
Si	1.17	1.71	1.91
SiC	2.42	2.82	2.86
ZnS	3.84	5.11	4.60
ZnSe	2.82	4.58	4.35

TABLE S22: Mean absolute errors (MAEs) for the lattice constants (*a*), band gaps (BG), and bulk moduli (BM) of the SS20 dataset obtained with conventional PBE0-D3(ABC) periodic calculations using the sol-def2-mSVP and pob-TZVP-rev2 basis sets.

PBE0-D3(ABC)	*a* (Å)	BG (eV)	BM (GPa)
sol-def2-mSVP	0.04	0.80	8.85
pob-TZVP-rev2	0.03	1.03	9.74

C. Graphite

TABLE S23: Experimental and predicted structural parameters and interlayer binding energy for Graphite, lattice parametes in Å, volume in Å^3 ed interlayer binding energy in meV/atom

	a	*c*	*V*	binding
Ref.[4,5]	2.458	6.700	34.900	-56.0
sol-def2-mSVP	2.455	6.642	34.700	-58.9
pob-TZVP-rev2	2.449	6.417	33.300	-69.5

The interlayer binding-energy profiles of graphite reveal that an accurate description of cohesion requires the full potential-energy surface to be assessed, rather than the binding energy alone. Uncorrected PBE0-D3(ABC) calculations with both sol-def2-mSVP and pob-TZVP-rev2 basis sets produce apparently favourable well depths, but these are strongly affected by basis-set superposition error. Counterpoise correction substantially reduces the binding strength and reshapes the interaction curve, demonstrating that BSSE is distance-dependent and cannot be regarded as a simple energetic offset. This is particularly critical for graphite, where the equilibrium interlayer spacing results from a delicate balance between Pauli repulsion and dispersion attraction. Apparent agreement with experimental or Quantum Monte Carlo reference energies[4] may therefore arise from fortuitous error cancellation while still yielding an inaccurate interaction potential. In contrast, the r^{2}SCAN-based composite schemes incorporate basis-set incompleteness and BSSE corrections directly into the energy expression, providing internally consistent potential-energy surfaces for both structural optimization and cohesive energetics. Both r^{2}SCAN0pob-3c and r^{2}SCAN0sol-3c predict shallower binding wells, close to the experimental equilibrium region. Overall, these results highlight that reliable modelling of graphite cohesion and equilibrium geometry demands a balanced and consistent treatment of basis-set effects, BSSE and dispersion rather than the adopted functional.

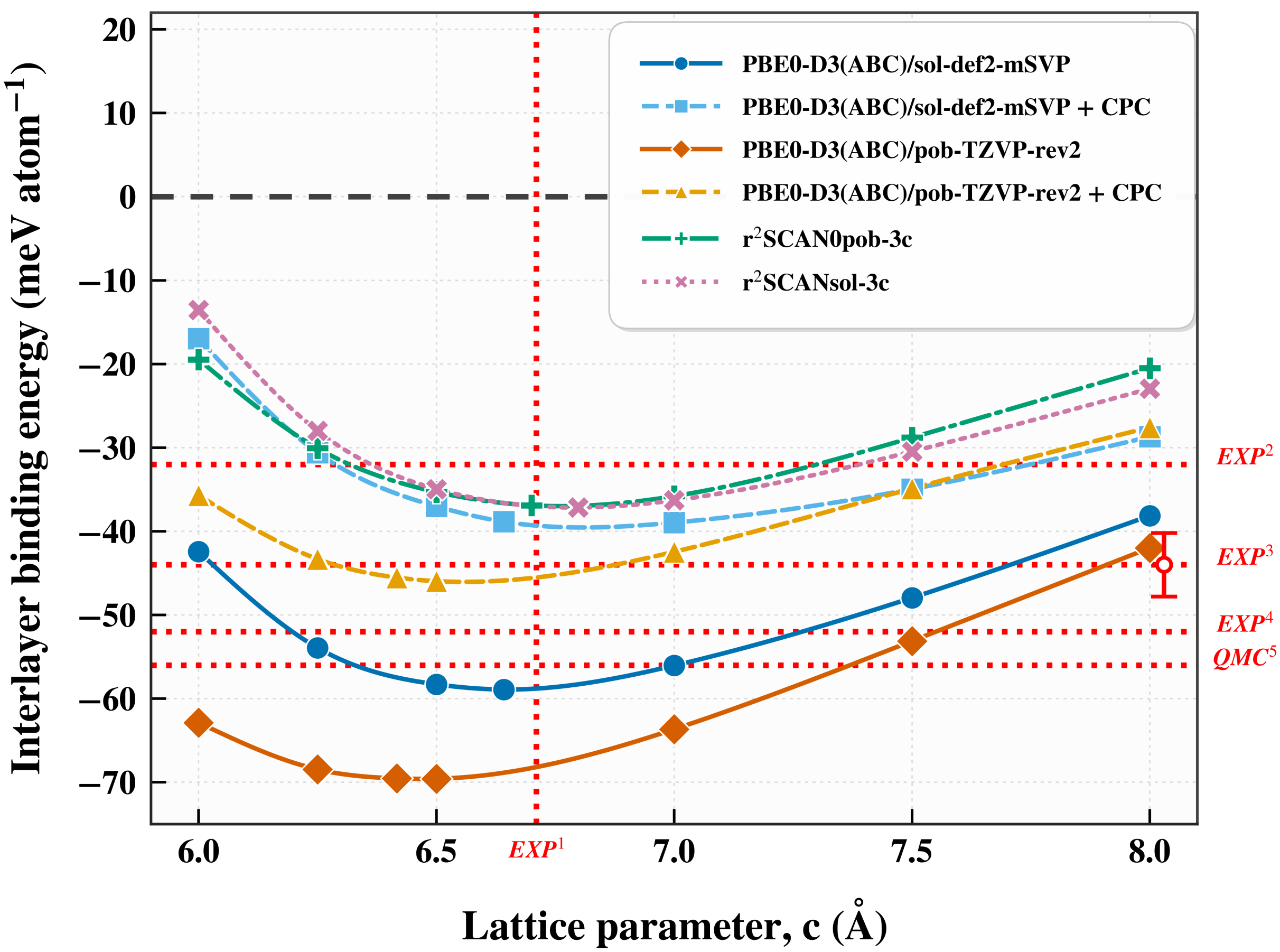

FIG. S2: Interlayer binding-energy landscape as a function of the out-of-plane lattice parameter. Interlayer binding energy per atom, E_{bind}, calculated as a function of the lattice parameter c using PBE0-D3(ABC) with the sol-def2-mSVP and pob-TZVP-rev2 basis sets, both with and without counterpoise correction (CPC), and the composite r^2SCAN0pob-3c and r^2SCANsol-3c methods. Energies are reported in meV atom^{-1} and c in Å. Dashed lines denote literature reference values: EXP[1] ([5]), EXP[2] ([24]), EXP[3] ([25]), EXP[4] ([26]) and QMC[5] ([4])

REFERENCES

[1] G. A. Dolgonos, J. Hoja, and A. D. Boese, "Revised values for the x23 benchmark set of molecular crystals," *Physical Chemistry Chemical Physics*, vol. 21, no. 44, pp. 24333–24344, 2019.

[2] A. Zen, J. G. Brandenburg, J. Klimeš, A. Tkatchenko, D. Alfè, and A. Michaelides, "Fast and accurate quantum monte carlo for molecular crystals," *Proceedings of the National Academy of Sciences*, vol. 115, no. 8, pp. 1724–1729, 2018.

[3] P. Pernot, B. Civalleri, D. Presti, and A. Savin, "Prediction uncertainty of density functional approximations for properties of crystals with cubic symmetry," *J. Phys. Chem. A*, vol. 119, pp. 5288–5304, 2015.

[4] L. Spanu, S. Sorella, and G. Galli, "Nature and strength of interlayer binding in graphite," *Physical review letters*, vol. 103, no. 19, p. 196401, 2009.

[5] Y. Baskin and L. Meyer, "Lattice constants of graphite at low temperatures," *Physical Review*, vol. 100, no. 2, p. 544, 1955.

[6] L. Liu, Y. Feng, and Z. Shen, "Structural and electronic properties of h-bn," *Physical Review B*, vol. 68, no. 10, p. 104102, 2003.

[7] C.-R. Hsing, C. Cheng, J.-P. Chou, C.-M. Chang, and C.-M. Wei, "Van der waals interaction in a boron nitride bilayer," *New Journal of Physics*, vol. 16, no. 11, p. 113015, 2014.

[8] M. Novotnỳ, M. Dubeckỳ, and F. Karlickỳ, "Toward accurate modeling of structure and energetics of bulk hexagonal boron nitride," *Journal of Computational Chemistry*, vol. 45, no. 2, pp. 115–121, 2024.

[9] A. Brown and S. Rundqvist, "Refinement of the crystal structure of black phosphorus," *Acta Crystallographica*, vol. 19, no. 4, pp. 684–685, 1965.

[10] G. Sansone, L. Maschio, D. Usvyat, M. Schutz, and A. Karttunen, "Toward an accurate estimate of the exfoliation energy of black phosphorus: a periodic quantum chemical approach," *The journal of physical chemistry letters*, vol. 7, no. 1, pp. 131–136, 2016.

[11] G. Sansone, A. J. Karttunen, D. Usvyat, M. Schütz, J. G. Brandenburg, and L. Maschio, "On the exfoliation and anisotropic thermal expansion of black phosphorus," *Chemical Communications*, vol. 54, no. 70, pp. 9793–9796, 2018.

[12] C. M. Zicovich-Wilson, F. Pascale, C. Roetti, V. Saunders, R. Orlando, and R. Dovesi, "Calculation of the vibration frequencies of α-quartz: The effect of hamiltonian and basis set," *Journal of computational chemistry*, vol. 25, no. 15, pp. 1873–1881, 2004.

[13] B. Xiao, J. Sun, A. Ruzsinszky, J. Feng, R. Haunschild, G. E. Scuseria, and J. P. Perdew, "Testing density functionals for structural phase transitions of solids under pressure: Si, sio 2, and zr," *Physical Review B—Condensed Matter and Materials Physics*, vol. 88, no. 18, p. 184103, 2013.

[14] M. Cutini, B. Civalleri, and P. Ugliengo, "Cost-effective quantum mechanical approach for predicting thermodynamic and mechanical stability of pure-silica zeolites," *ACS Omega*, vol. 4, no. 1, pp. 1838–1846, 2019.

[15] P. Ugliengo and A. Damin, "Are dispersive forces relevant for co adsorption on the mgo (0 0 1) surface?," *Chemical physics letters*, vol. 366, no. 5-6, pp. 683–690, 2002.

[16] R. Valero, J. R. Gomes, D. G. Truhlar, and F. Illas, "Good performance of the m06 family of hybrid meta generalized gradient approximation density functionals on a difficult case: Co adsorption on mgo (001)," *The Journal of chemical physics*, vol. 129, no. 12, 2008.

[17] B. Civalleri, L. Maschio, P. Ugliengo, and C. M. Zicovich-Wilson, "Role of dispersive interactions in the co adsorption on mgo (001): periodic b3lyp calculations augmented with an empirical dispersion term," *Physical Chemistry Chemical Physics*, vol. 12, no. 24, pp. 6382–6386, 2010.

[18] V. Staemmler, "Method of local increments for the calculation of adsorption energies of atoms and small molecules on solid surfaces. 2. co/mgo (001)," *The Journal of Physical Chemistry A*, vol. 115, no. 25, pp. 7153–7160, 2011.

[19] A. D. Boese and J. Sauer, "Accurate adsorption energies of small molecules on oxide surfaces: Co–mgo (001)," *Physical chemistry chemical physics*, vol. 15, no. 39, pp. 16481–16493, 2013.

[20] M. Alessio, D. Usvyat, and J. Sauer, "Chemically accurate adsorption energies: Co and h2o on the mgo (001) surface," *Journal of Chemical Theory and Computation*, vol. 15, no. 2, pp. 1329–1344, 2018.

[21] S. K. Dunn and G. E. Ewing, "Infrared spectra and structure of acetylene on sodium chloride (100)," *The Journal of Physical Chemistry*, vol. 96, no. 13, pp. 5284–5290, 1992.

[22] A. Cabello-Cartagena, J. Vogt, and H. Weiss, "Structure and infrared absorption of the first layer c2h2 on the nacl (100) single-crystal surface," *The Journal of chemical physics*, vol. 132, no. 7, 2010.

[23] L. Valenzano, B. Civalleri, K. Sillar, and J. Sauer, "Heats of adsorption of co and co2 in metal–organic frameworks: quantum mechanical study of cpo-27-m (m= mg, ni, zn)," *The Journal of Physical Chemistry C*, vol. 115, no. 44, pp. 21777–21784, 2011.

[24] L. X. Benedict, N. G. Chopra, M. L. Cohen, A. Zettl, S. G. Louie, and V. H. Crespi, "Microscopic determination of the interlayer binding energy in graphite," *Chemical Physics Letters*, vol. 286, no. 5-6, pp. 490–496, 1998.

[25] J. Weippert, J. Hauns, J. Bachmann, A. Böttcher, X. Yao, B. Yang, A. Narita, K. Müllen, and M. M. Kappes, "A tpd-based determination of the graphite interlayer cohesion energy," *The Journal of Chemical Physics*, vol. 149, p. 194701, 11 2018.

[26] R. Zacharia, H. Ulbricht, and T. Hertel, "Interlayer cohesive energy of graphite from thermal desorption of polyaromatic hydrocarbons," *Physical Review B*, vol. 69, no. 15, p. 155406, 2004.
